# A stable gene selection in microarray data analysis

**DOI:** 10.1186/1471-2105-7-228

**Published:** 2006-04-27

**Authors:** Kun Yang, Zhipeng Cai, Jianzhong Li, Guohui Lin

**Affiliations:** 1 Department of Computer Science and Engineering, Harbin Institute of Technology, Harbin 150001, China; 2 Department of Computing Science, University of Alberta. Edmonton, Alberta T6G 2E8, Canada

## Abstract

**Background:**

Microarray data analysis is notorious for involving a huge number of genes compared to a relatively small number of samples. Gene selection is to detect the most significantly differentially expressed genes under different conditions, and it has been a central research focus. In general, a better gene selection method can improve the performance of classification significantly. One of the difficulties in gene selection is that the numbers of samples under different conditions vary a lot.

**Results:**

Two novel gene selection methods are proposed in this paper, which are not affected by the unbalanced sample class sizes and do not assume any explicit statistical model on the gene expression values. They were evaluated on eight publicly available microarray datasets, using leave-one-out cross-validation and 5-fold cross-validation. The performance is measured by the classification accuracies using the top ranked genes based on the training datasets.

**Conclusion:**

The experimental results showed that the proposed gene selection methods are efficient, effective, and robust in identifying differentially expressed genes. Adopting the existing SVM-based and KNN-based classifiers, the selected genes by our proposed methods in general give more accurate classification results, typically when the sample class sizes in the training dataset are unbalanced.

## Background

DNA microarray is a technology that can simultaneously measure the expression levels of thousands of genes in a single experiment. It is commonly used for comparing the gene expression levels in tissues under different conditions, such as wild-type versus mutant, or healthy versus diseased [1]. Some of the genes are expected to be differentially modulated in tissues under different conditions, with their expression levels increased or decreased to signify the experimental conditions. These discriminatory genes are very useful in clinical applications such as recognizing diseased profiles. However, due to high cost, the number of experiments that can be used for classification purpose is usually limited. This small number of experiments, compared to the large number of genes in an experiment, wakes up "the curse of dimensionality" and challenges the classification task and other data analysis in general. It is well-known that quite a number of genes are house-keeping genes and many others could be unrelated to the classification task [2]. Therefore, an important step to effective classification is to identify the discriminatory genes thus to reduce the number of genes used for classification purpose. This step of discriminatory gene identification is generally referred to as *gene selection*. Gene selection is a pre-requisite in many applications [3]. It should be noted that, often, the number of unrelated genes is much larger than the number of discriminatory genes.

There are a variety of gene selection methods proposed in the last a few years [2,4,5]. Among them, some methods assume explicit statistical models on the gene expression data. For example, Baldi and Long [4] developed a Gaussian gene-independent model on the gene expression data, and implemented a t-test combined with a full Bayesian treatment for gene selection. These methods assuming certain models are referred to as *model-based *gene selection methods. Other methods do not assume any specific distribution model on the gene expression data and they are referred to as *model-free *gene selection methods. For example, Xiong et al. [2] suggested a method to select genes through the space of feature subsets using classification errors. Guyon et al. [5] proposed a gene selection approach utilizing support vector machines based on recursive feature elimination. It has been reported that the results of model-free gene selection methods may be influenced by the classification methods chosen for scoring the genes [6]. Nonetheless, model-based gene selection methods lack of adaptability, because it is unlikely possible to construct a universal probabilistic analysis model that is suitable for all kinds of gene expression data, where noise and variance may vary dramatically across different gene expression data [6]. In this sense, model-free gene selection methods are more desirable than model-based ones.

Gene selection is to provide a subset of a small number of discriminatory, or the most relevant, genes that can effectively recognize the class to which a testing sample belongs. That is, it is to provide a classifier such that the classification error is minimized. The known dataset that is used for learning the classifier, or equivalently for gene selection, is referred to as the *training dataset*. In a training dataset, every sample is labeled with its known class. Notice that if one class is significantly larger than the others, then the trained classifier might be biased. Therefore, the desired gene selection methods are those that are not affected by the sizes of classes in the training dataset. A gene selection method is called *stable *if the selected genes are kept the same when duplicating all the samples in any class in the training dataset.

In this paper, we propose two novel gene scoring functions *s*_1_(·) and *s*_2_(·) to design two stable gene selection methods GS1 and GS2 [see Additional file 5], respectively, to be detailed in the Methods section. According to the classification scheme proposed in [6], our proposed gene selection methods are single gene scoring approaches. These two gene scoring functions non-trivially incorporate the means and the variations of the expression values of genes in the samples belonging to a common class, based on a very general assumption that discriminatory genes are those having different means across different classes, small *intra-class variations *and relatively large *inter-class variations*. This spherical data assumption does not involve any specific statistical model, and in this sense, the resultant gene selection methods GS1 and GS2 could be regarded as model-free. They are also shown to be stable theoretically.

## Results and discussion

We have applied our gene selection methods GS1 and GS2 based on the gene scoring functions *s*_1_(·) and *s*_2_(·), respectively, to a total of 8 publicly available microarray datasets [7]: the *leukemia *(LEU) dataset [8], the *small round blue cell tumors *(SRBCT) dataset [9], the *malignant glioma *(GLIOMA) dataset [10], the *human lung carcinomas *(LUNG) dataset [11], the *human carcinomas *(CAR) dataset [12], the *mixed-lineage leukemia *(MLL) dataset [13], the *prostate *(PROSTATE) dataset [14], and the *diffuse large B-cell lymphoma *(DLBCL) dataset [15], the first two of which have been used for several similar testings of gene selection methods. On each dataset, one portion was used as the training dataset for our methods to score the genes and the other portion was used as the testing dataset. For each specified number *x *we reported the classification accuracy, on the testing dataset, of the classifier based on the top ranked *x *genes using the training dataset. The quality of these top ranked *x *genes is justified on two aspects: 1) the classification accuracy of the resultant classifier on the testing datasets, and 2) for the first two datasets LEU and SRBCT, the stability when the training datasets were partially changed. All the experiments were conducted in MATLAB [16] environment on a Pentium IV PC with a 2.4 GHz processor and a 512 MB RAM.

### The datasets

The LEU dataset contains in total 72 samples in two classes, *acute lymphoblastic leukemia *(ALL) and *acute myeloid leukemia *(AML), which contain 47 and 25 samples, respectively. Every sample contains 7,129 gene expression values. We adopted the pretreatment method proposed in [1] to remove about half the genes and subsequently every sample contains only 3, 571 gene expression values.

The SRBCT dataset contains in total 83 samples in four classes, *the Ewing family of tumors *(EWS), *Burkitt lymphoma *(BL), *neuroblastoma *(NB) and *rhabdomyosarcoma *(RMS) [9]. Every sample in this dataset contains only 2,308 gene expression values. Among the 83 samples, 29, 11, 18, and 25 samples belong to classes EWS, BL, NB and RMS, respectively.

The GLIOMA dataset [10] contains in total 50 samples in four classes, *cancer glioblastomas *(CG), *non-cancer glioblastomas *(NG), *cancer oligodendrogliomas *(CO) and *non-cancer oligodendrogliomas *(NO), which have 14,14, 7,15 samples, respectively. Each sample has 12625 genes. We adopted a standard filtering method [10], that is, genes with minimal variations across the samples were removed. For this dataset, intensity thresholds were set at 20 and 16,000 units. Genes whose expression levels varied < 100 units between samples, or varied < 3 fold between any two samples, were excluded. After preprocessing, we obtained a dataset with 50 samples and 4433 genes.

The LUNG dataset [11] contains in total 203 samples in five classes, *adenocarcinomas, squamous cell lung carcinomas, pulmonary carcinoids, small-cell lung carcinomas *and *normal lung*, which have 139, 21, 20, 6,17 samples, respectively. Each sample has 12600 genes. The genes with standard deviations smaller than 50 expression units were removed and we obtained a dataset with 203 samples and 3312 genes [11].

The CAR dataset [12] contains in total 174 samples in eleven classes, *prostate, bladder/ureter, breast, colorectal, gastroesophagus, kidney, liver, ovary, pancreas, lung adenocarcinomas*, and *lung squamous cell carcinoma*, which have 26, 8, 26, 23,12,11, 7, 27, 6,14,14 samples, respectively. Each sample contains 12533 genes. In our experiment, we preprocessed dataset as described in [12]. We included only those probe sets whose maximum hybridization intensity (AD) in at least one sample was 200, all AD values ≤ 20, including negative AD values, were raised to 20, and the data were log transformed. After preprocessing, we obtained a dataset with 174 samples and 9182 genes.

The MLL dataset [13] contains in total 72 samples in three classes, *acute lymphoblastic leukemia *(ALL), *acute myeloid leukemia *(AML), and *mixed-lineage leukemia gene *(MLL), which have 24, 28, 20 samples, respectively. In our experiment, intensity thresholds were set at 100 – 16000 units. Then the relative variation of expressions for each gene was determined by dividing the maximum expression for the gene among all samples (max) over the minimum expression (min), i.e. max/min. We filtered out the genes with max/min ≤ 5 or (max - min) ≤ 500, that is, for max/min fold variation, we excluded genes with less than 5-fold variation and, for (max - min) absolute variation, we excluded genes with less than 500 units absolute. After preprocessing, we obtained a dataset with 72 samples and 8685 genes.

The PROSTATE dataset [14] contains in total 102 samples in two classes *tumor *and *normal*, which have 52 and 50 samples, respectively. The original dataset contains 12600 genes. In our experiment, intensity thresholds were set at 100 – 16000 units, the same as in the MLL dataset. Then we filtered out the genes with max/min ≤ 5 or (max - min) ≤ 50. After preprocessing, we obtained a dataset with 102 samples and 5966 genes.

The DLBCL dataset [15] contains in total 77 samples in two classes, *diffuse large B-cell lymphomas *(DLBCL) and *follicular lymphoma *(FL) which have 58 and 19 samples, respectively. The original dataset contains 7129 genes. We set intensity thresholds at 20 – 16000 units, then we filtered out genes with max/min ≤ 3 or (max - min) ≤ 100. After preprocessing, we obtained a dataset with 77 samples and 6285 genes.

Among the above 8 datasets [see Additional files 3 and 4], the first two, LEU and SRBCT, have been used frequently for testing gene selection methods and classifiers. For each of them, if we use the ratio of the largest class size divided by the smallest class size to represent the level of unbalance, then the fifth dataset CAR is the most unbalanced. In our experiments, we have run every gene selection method on each of the 8 datasets to rank the genes, and for every *x *≤ 100, the classification accuracies of the classifier built using the top ranked *x *genes have been collected [see Additional file 1]. We chose to present part of the classification accuracies on datasets SRBCT and CAR in details (as plots) and to present only three values for *x*, 30, 60, and 100, for all eight datasets (as tables).

### Classification accuracies

Using the top ranked genes selected by a gene selection method, together with their expression values in the training dataset, one can build a classifier that will decide for each testing sample the class it belongs to. Only the expression values for those selected genes in the testing sample are used for such a decision making. This is a standard way to test the quality of those selected genes, to examine how well the resulting classifier performs. Note that testing samples are not included in the training dataset. To this purpose, we define the *classification accuracy *to be the percentage of the correct decisions made by the classifier on the testing samples. We have compared our methods GS1 and GS2 with two other model-free gene selection methods Cho's [17] and F-test [1]. In our experiments, we have adopted two ways to build a classifier using the selected genes, one is through Support Vector Machines (SVMs) [5] and the other is through *K-*Nearest-Neighbor (KNN) search [1]. Essentially, SVMs compute a decision plane to separate the set of chips (in the training dataset) having different class memberships, and use this plane to predict the class memberships for testing chips. There are a number of kernels used in SVMs models for decision plane computing and we chose a linear kernel as described in [5]. A KNN classifier ascertains the class of a testing sample by analyzing its *K *nearest neighbors in the training dataset [1]. We chose the Euclidean distance in our KNN classifier with *K *= 5 and predicted the class by majority vote [1]. The SVM we used in MATLAB is from [18] and we coded the KNN by ourselves. For ease of presentation, the achieved classifiers are referred to as the SVM-classifier and the KNN-classifier, respectively.

**Figure 1:**
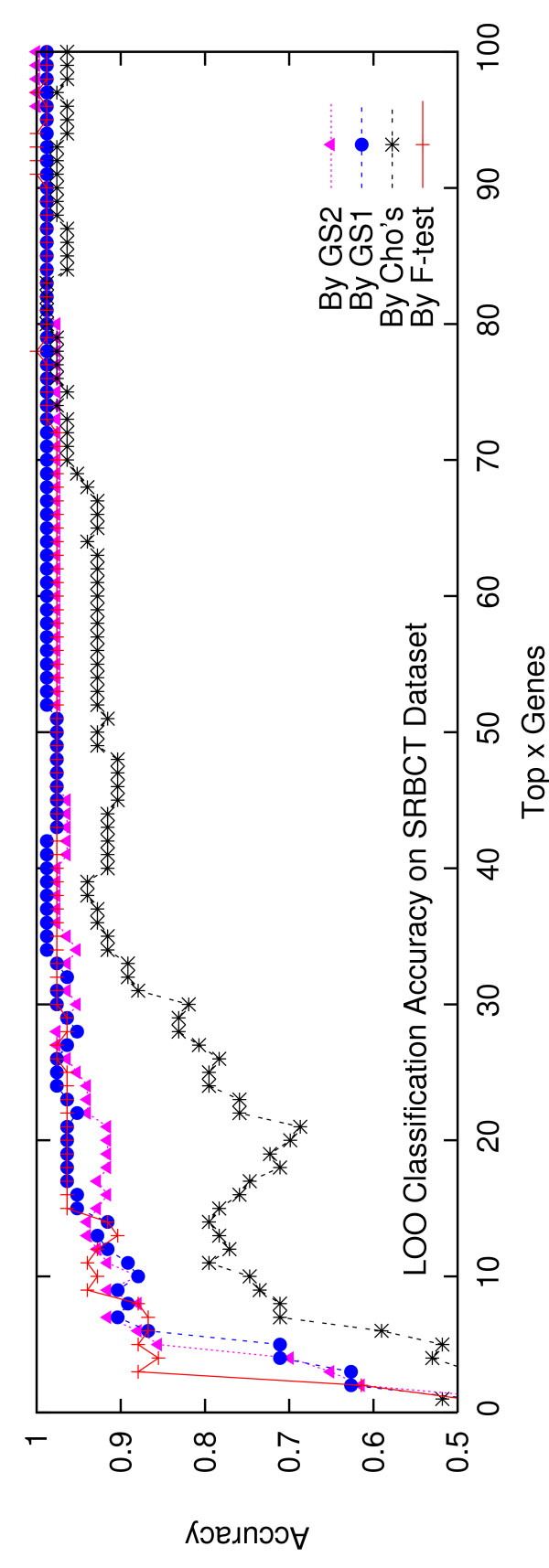
The leave-one-out cross validation classification accuracies of the SVM-classifier on four gene selection methods, GS1, GS2, Cho's, and F-test, on the SRBCT dataset.

**Figure 2:**
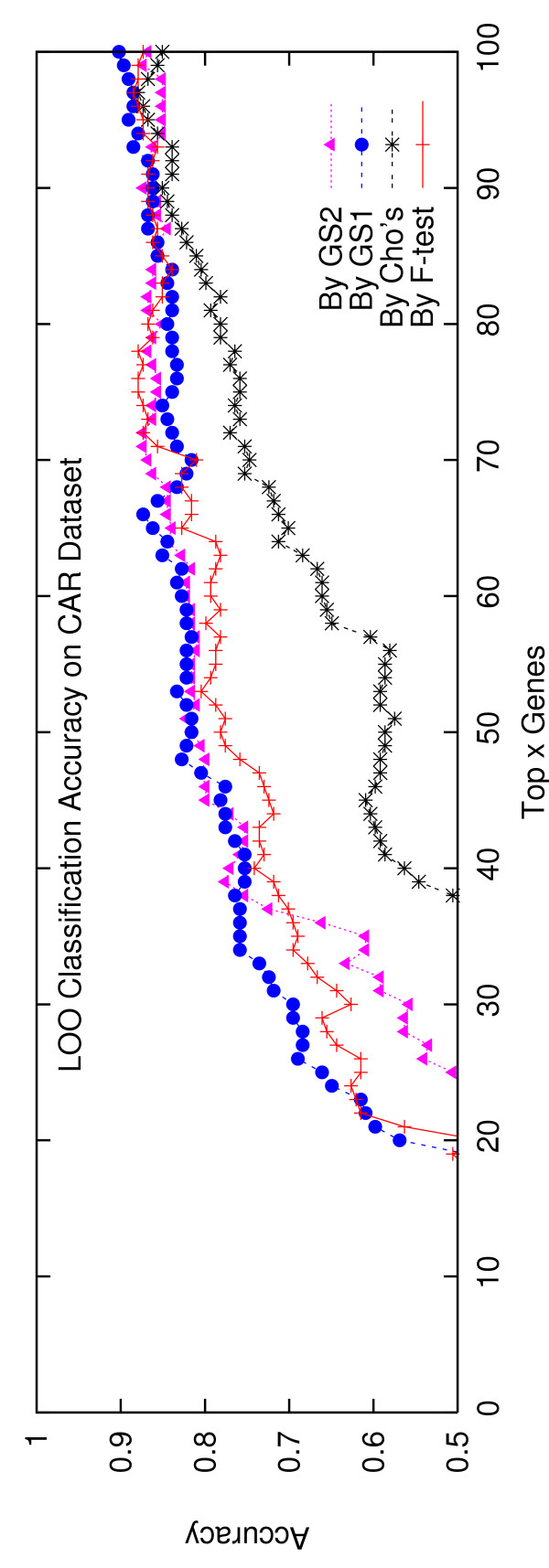
The leave-one-out cross validation classification accuracies of the SVM-classifier on four gene selection methods, GS1, GS2, Cho's, and F-test, on the CAR dataset.

Figures 1 and 2 plot the classification accuracies of the SVM-classifier based on four gene selection methods GS1, GS2, Cho's, and F-test, on the SRBCT and CAR datasets, respectively. These classification accuracies were obtained through *Leave-One-Out *(LOO) cross validation. In LOO cross validation, one sample was left out as a testing sample and the remaining were used as the training dataset, and this was done for every sample in the dataset. We have also conducted 5-Fold cross validation, in which each dataset was randomly partitioned into 5 parts of equal size and in every experiment four parts were used as the training dataset (the fifth part was used as the testing dataset). This was done for every four parts in the dataset and the process (that is, random partition, training, and testing) was repeated for 100 times. The average accuracy over all these 500 testing datasets was taken as the 5-Fold cross validation classification accuracy. All plots of the 5-Fold (and the other LOO) cross validation classification accuracies of the SVM-classifier and the KNN-classifier based on four gene selection methods GS1, GS2, Cho's, and F-test, on the eight datasets are included in Additional file 1. Especially, columns 2-4 (and 6–8) in Tables 1, 2, 3, 4, 5, 6, 7, 8 record these cross validation classification accuracies, for only three numbers of top ranked genes, that is, 30, 60, and 100. Column 5 (and column 9) records the highest cross validation classification accuracies on these eight datasets ever achieved by the SVM-classifier and the KNN-classifier, respectively, as well as the associated numbers of selected genes (no more than 100 genes were used).

Note that in the 5-fold cross validation, the classification accuracy is calculated as the average of 500 classification accuracies on 500 testing datasets. We have also collected their standard deviations [see Additional file 2]. For three numbers 30, 60, and 100, the standard deviations are included in Tables 1, 2, 3, 4, 5, 6, 7, 8. Essentially, all these four gene selection methods, GS1, GS2, Cho's, and F-test, have very close standard deviations, and these standard deviations seem to be independent of classifier and dataset. Consequently, looking at all the classification accuracies as shown in Figures 1, 2 and Tables 1, 2, 3, 4, 5, 6, 7, 8, one general conclusion is that our gene selection methods, GS1 and GS2, perform at least comparably well to F-test and Cho's, on all 8 datasets using both the SVM-classifier and the KNN-classifier. Typically, our methods outperform significantly the other two methods on datasets SRBCT, GLIOMA, LUNG, and CAR, which have unbalanced class sizes.

### Stability of the gene selection methods

Given a training dataset (in this case, we take the whole dataset as the training dataset), to test the stability of a gene selection method we duplicated all the samples in one class to produce a *duplicated *dataset. Our gene selection methods GS1 and GS2 are shown to be stable theoretically (cf. Methods section) and therefore are not subjects to such a test. For each of Cho's and F-test, it was applied on the duplicated datasets to report the same numbers of genes as it was applied to the original training dataset, and then the percentages of the common genes were recorded. Note that the LEU dataset and the SRBCT dataset give 2 and 4 duplicated datasets, respectively. Table 9 collects these percentages.

We have also performed a similar experiment to test the stability when a small portion of the samples were removed from the training dataset. For each class in a training dataset, it was divided into three parts of equal size and every time one part was removed from the dataset to obtain a *reduced *dataset. Then again, the percentages of the common selected genes using the original dataset and the reduced datasets were recorded. We tried in total 1000 random divisions and the average of 3000 percentages was taken to be the stability for this class. Table 10 collects these stability results for GS1, GS2, Cho's, and F-test. From these results, we can see that GS1, GS2, and F-test had very close stabilities on the reduced datasets, while Cho's had the least over all classes.

## Conclusion

In this paper, we proposed two stable gene selection methods GS1 and GS2, which could also be regarded as model-free. From the comparisons made to one most recent method Cho's and one most classic method F-test on eight publicly available datasets, GS1 and GS2 selected genes whose resultant classifiers achieved at least equally good and most of the time better classification accuracies. Both leave-one-out and 5-fold cross validations confirmed our conclusion. We haven't run any biological experiments to verify each of the top ranked genes by our methods yet inconsistent to other methods. Nonetheless, we believe that our methods would be good potential substitutes to the ones currently in use as our methods are model-free and stable.

## Methods

Assume in the training dataset there are in total *p *genes in the microarray chips, and assume we have in total *n *chips/samples that have been grouped into *L *classes. Let *a*_*ij *_denote the expression value of gene *j *in sample *i*. The training dataset can thus be represented as a matrix

*A*_*n*×*p *_= (*a*_*ij*_)_*n*×*p*_.

We will define two gene scoring functions using entry values in matrix *A*_*n*×*p*_. These two scoring functions might be considered to better use the means and the variations of the gene expression values.

Let *n*_*k *_denote the number of samples in class *C*_*k*_, for *k *= 1, 2,..., *L *(i.e., ∑k=1Lnk=n
 MathType@MTEF@5@5@+=feaafiart1ev1aaatCvAUfKttLearuWrP9MDH5MBPbIqV92AaeXatLxBI9gBaebbnrfifHhDYfgasaacH8akY=wiFfYdH8Gipec8Eeeu0xXdbba9frFj0=OqFfea0dXdd9vqai=hGuQ8kuc9pgc9s8qqaq=dirpe0xb9q8qiLsFr0=vr0=vr0dc8meaabaqaciaacaGaaeqabaqabeGadaaakeaadaaeWaqaaiabd6gaUnaaBaaaleaacqWGRbWAaeqaaOGaeyypa0JaemOBa4galeaacqWGRbWAcqGH9aqpcqaIXaqmaeaacqWGmbata0GaeyyeIuoaaaa@3889@). Let a¯kj=1nk∑i∈Ckaij
 MathType@MTEF@5@5@+=feaafiart1ev1aaatCvAUfKttLearuWrP9MDH5MBPbIqV92AaeXatLxBI9gBaebbnrfifHhDYfgasaacH8akY=wiFfYdH8Gipec8Eeeu0xXdbba9frFj0=OqFfea0dXdd9vqai=hGuQ8kuc9pgc9s8qqaq=dirpe0xb9q8qiLsFr0=vr0=vr0dc8meaabaqaciaacaGaaeqabaqabeGadaaakeaacuWGHbqygaqeamaaBaaaleaacqWGRbWAcqWGQbGAaeqaaOGaeyypa0ZaaSqaaSqaaiabigdaXaqaaiabd6gaUnaaBaaameaacqWGRbWAaeqaaaaakmaaqababaGaemyyae2aaSbaaSqaaiabdMgaPjabdQgaQbqabaaabaGaemyAaKMaeyicI4Saem4qam0aaSbaaWqaaiabdUgaRbqabaaaleqaniabggHiLdaaaa@419A@, which is the average expression value of gene *j *in class *C*_*k*_, for *k *= 1, 2,..., *L*. The expression vector (a¯k1,a¯k2,…,a¯kp
 MathType@MTEF@5@5@+=feaafiart1ev1aaatCvAUfKttLearuWrP9MDH5MBPbIqV92AaeXatLxBI9gBaebbnrfifHhDYfgasaacH8akY=wiFfYdH8Gipec8Eeeu0xXdbba9frFj0=OqFfea0dXdd9vqai=hGuQ8kuc9pgc9s8qqaq=dirpe0xb9q8qiLsFr0=vr0=vr0dc8meaabaqaciaacaGaaeqabaqabeGadaaakeaacuWGHbqygaqeamaaBaaaleaacqWGRbWAcqaIXaqmaeqaaOGaeiilaWIafmyyaeMbaebadaWgaaWcbaGaem4AaSMaeGOmaidabeaakiabcYcaSiablAciljabcYcaSiqbdggaHzaaraWaaSbaaSqaaiabdUgaRjabdchaWbqabaaaaa@3C97@) is the *centroid *of class *C*_*k*_. Correspondingly, the centroid matrix is

A¯L×p=(a¯kj)L×p.
 MathType@MTEF@5@5@+=feaafiart1ev1aaatCvAUfKttLearuWrP9MDH5MBPbIqV92AaeXatLxBI9gBaebbnrfifHhDYfgasaacH8akY=wiFfYdH8Gipec8Eeeu0xXdbba9frFj0=OqFfea0dXdd9vqai=hGuQ8kuc9pgc9s8qqaq=dirpe0xb9q8qiLsFr0=vr0=vr0dc8meaabaqaciaacaGaaeqabaqabeGadaaakeaacuWGbbqqgaqeamaaBaaaleaacqWGmbatcqGHxdaTcqWGWbaCaeqaaOGaeyypa0JaeiikaGIafmyyaeMbaebadaWgaaWcbaGaem4AaSMaemOAaOgabeaakiabcMcaPmaaBaaaleaacqWGmbatcqGHxdaTcqWGWbaCaeqaaOGaeiOla4caaa@3F6E@

The mean of these centroids is A^=(a^1,a^2,…,a^p)
 MathType@MTEF@5@5@+=feaafiart1ev1aaatCvAUfKttLearuWrP9MDH5MBPbIqV92AaeXatLxBI9gBaebbnrfifHhDYfgasaacH8akY=wiFfYdH8Gipec8Eeeu0xXdbba9frFj0=OqFfea0dXdd9vqai=hGuQ8kuc9pgc9s8qqaq=dirpe0xb9q8qiLsFr0=vr0=vr0dc8meaabaqaciaacaGaaeqabaqabeGadaaakeaacuWGbbqqgaqcaiabg2da9maabmaabaGafmyyaeMbaKaadaWgaaWcbaGaeGymaedabeaakiabcYcaSiqbdggaHzaajaWaaSbaaSqaaiabikdaYaqabaGccqGGSaalcqWIMaYscqGGSaalcuWGHbqygaqcamaaBaaaleaacqWGWbaCaeqaaaGccaGLOaGaayzkaaaaaa@3C16@, where a^j=1L∑k=1La¯kj
 MathType@MTEF@5@5@+=feaafiart1ev1aaatCvAUfKttLearuWrP9MDH5MBPbIqV92AaeXatLxBI9gBaebbnrfifHhDYfgasaacH8akY=wiFfYdH8Gipec8Eeeu0xXdbba9frFj0=OqFfea0dXdd9vqai=hGuQ8kuc9pgc9s8qqaq=dirpe0xb9q8qiLsFr0=vr0=vr0dc8meaabaqaciaacaGaaeqabaqabeGadaaakeaacuWGHbqygaqcamaaBaaaleaacqWGQbGAaeqaaOGaeyypa0ZaaSqaaSqaaiabigdaXaqaaiabdYeambaakmaaqadabaGafmyyaeMbaebadaWgaaWcbaGaem4AaSMaemOAaOgabeaaaeaacqWGRbWAcqGH9aqpcqaIXaqmaeaacqWGmbata0GaeyyeIuoaaaa@3D8F@.

For sample *i *belonging to class *C*_*k*_, the difference between the expression value of gene *j *and the class mean is *x*_*ij *_= |*a*_*ij *_- a¯kj
 MathType@MTEF@5@5@+=feaafiart1ev1aaatCvAUfKttLearuWrP9MDH5MBPbIqV92AaeXatLxBI9gBaebbnrfifHhDYfgasaacH8akY=wiFfYdH8Gipec8Eeeu0xXdbba9frFj0=OqFfea0dXdd9vqai=hGuQ8kuc9pgc9s8qqaq=dirpe0xb9q8qiLsFr0=vr0=vr0dc8meaabaqaciaacaGaaeqabaqabeGadaaakeaacuWGHbqygaqeamaaBaaaleaacqWGRbWAcqWGQbGAaeqaaaaa@30F7@|. The matrix

*X*_*n*×*p *_= (*x*_*ij*_)_*n*×*p*_

is the *deviation matrix *of the training dataset. Let x¯kj=1nk∑i∈Ckxij
 MathType@MTEF@5@5@+=feaafiart1ev1aaatCvAUfKttLearuWrP9MDH5MBPbIqV92AaeXatLxBI9gBaebbnrfifHhDYfgasaacH8akY=wiFfYdH8Gipec8Eeeu0xXdbba9frFj0=OqFfea0dXdd9vqai=hGuQ8kuc9pgc9s8qqaq=dirpe0xb9q8qiLsFr0=vr0=vr0dc8meaabaqaciaacaGaaeqabaqabeGadaaakeaacuWG4baEgaqeamaaBaaaleaacqWGRbWAcqWGQbGAaeqaaOGaeyypa0ZaaSqaaSqaaiabigdaXaqaaiabd6gaUnaaBaaameaacqWGRbWAaeqaaaaakmaaqababaGaemiEaG3aaSbaaSqaaiabdMgaPjabdQgaQbqabaaabaGaemyAaKMaeyicI4Saem4qam0aaSbaaWqaaiabdUgaRbqabaaaleqaniabggHiLdaaaa@41F6@ denote the average deviation for samples in class *C*_*k *_with respect to the centroid. The centroid deviation matrix is

X¯L×p=(x¯kj)L×p.
 MathType@MTEF@5@5@+=feaafiart1ev1aaatCvAUfKttLearuWrP9MDH5MBPbIqV92AaeXatLxBI9gBaebbnrfifHhDYfgasaacH8akY=wiFfYdH8Gipec8Eeeu0xXdbba9frFj0=OqFfea0dXdd9vqai=hGuQ8kuc9pgc9s8qqaq=dirpe0xb9q8qiLsFr0=vr0=vr0dc8meaabaqaciaacaGaaeqabaqabeGadaaakeaacuWGybawgaqeamaaBaaaleaacqWGmbatcqGHxdaTcqWGWbaCaeqaaOGaeyypa0JaeiikaGIafmiEaGNbaebadaWgaaWcbaGaem4AaSMaemOAaOgabeaakiabcMcaPmaaBaaaleaacqWGmbatcqGHxdaTcqWGWbaCaeqaaOGaeiOla4caaa@3FCA@

Intuitively, gene *j *has a strong discriminating power if the means a¯kj
 MathType@MTEF@5@5@+=feaafiart1ev1aaatCvAUfKttLearuWrP9MDH5MBPbIqV92AaeXatLxBI9gBaebbnrfifHhDYfgasaacH8akY=wiFfYdH8Gipec8Eeeu0xXdbba9frFj0=OqFfea0dXdd9vqai=hGuQ8kuc9pgc9s8qqaq=dirpe0xb9q8qiLsFr0=vr0=vr0dc8meaabaqaciaacaGaaeqabaqabeGadaaakeaacuWGHbqygaqeamaaBaaaleaacqWGRbWAcqWGQbGAaeqaaaaa@30F7@, *k *= 1, 2,..., *L*, differ significantly and x¯kj
 MathType@MTEF@5@5@+=feaafiart1ev1aaatCvAUfKttLearuWrP9MDH5MBPbIqV92AaeXatLxBI9gBaebbnrfifHhDYfgasaacH8akY=wiFfYdH8Gipec8Eeeu0xXdbba9frFj0=OqFfea0dXdd9vqai=hGuQ8kuc9pgc9s8qqaq=dirpe0xb9q8qiLsFr0=vr0=vr0dc8meaabaqaciaacaGaaeqabaqabeGadaaakeaacuWG4baEgaqeamaaBaaaleaacqWGRbWAcqWGQbGAaeqaaaaa@3125@, *k *= 1, 2,..., *L*, indicating the *intra-class variations*, are all small.

For example, suppose we have a microarray expression matrix *A*_12×4 _as shown, in which 12 samples have been known in 3 classes:

A12×4=(0.650.20.20.70.851.01.41.00.91.20.81.3¯0.90.70.60.51.11.42.00.71.51.81.21.61.30.91.01.2¯1.21.00.81.51.51.71.60.21.72.12.31.82.02.01.90.31.61.21.41.2).
 MathType@MTEF@5@5@+=feaafiart1ev1aaatCvAUfKttLearuWrP9MDH5MBPbIqV92AaeXatLxBI9gBaebbnrfifHhDYfgasaacH8akY=wiFfYdH8Gipec8Eeeu0xXdbba9frFj0=OqFfea0dXdd9vqai=hGuQ8kuc9pgc9s8qqaq=dirpe0xb9q8qiLsFr0=vr0=vr0dc8meaabaqaciaacaGaaeqabaqabeGadaaakeaacqWGbbqqdaWgaaWcbaGaeGymaeJaeGOmaiJaey41aqRaeGinaqdabeaakiabg2da9maabmaabaqbaeGabqqaaaaabaWaaWaaaeaafaqaceWaeaaaaeaacqaIWaamcqGGUaGlcqaI2aGncqaI1aqnaeaacqaIWaamcqGGUaGlcqaIYaGmaeaacqaIWaamcqGGUaGlcqaIYaGmaeaacqaIWaamcqGGUaGlcqaI3aWnaeaacqaIWaamcqGGUaGlcqaI4aaocqaI1aqnaeaacqaIXaqmcqGGUaGlcqaIWaamaeaacqaIXaqmcqGGUaGlcqaI0aanaeaacqaIXaqmcqGGUaGlcqaIWaamaeaacqaIWaamcqGGUaGlcqaI5aqoaeaacqaIXaqmcqGGUaGlcqaIYaGmaeaacqaIWaamcqGGUaGlcqaI4aaoaeaacqaIXaqmcqGGUaGlcqaIZaWmaaaaaaqaamaamaaabaqbaeqabqabaaaaaeaacqaIWaamcqGGUaGlcqaI5aqoaeaacqaIWaamcqGGUaGlcqaI3aWnaeaacqaIWaamcqGGUaGlcqaI2aGnaeaacqaIWaamcqGGUaGlcqaI1aqnaeaacqaIXaqmcqGGUaGlcqaIXaqmaeaacqaIXaqmcqGGUaGlcqaI0aanaeaacqaIYaGmcqGGUaGlcqaIWaamaeaacqaIWaamcqGGUaGlcqaI3aWnaeaacqaIXaqmcqGGUaGlcqaI1aqnaeaacqaIXaqmcqGGUaGlcqaI4aaoaeaacqaIXaqmcqGGUaGlcqaIYaGmaeaacqaIXaqmcqGGUaGlcqaI2aGnaeaacqaIXaqmcqGGUaGlcqaIZaWmaeaacqaIWaamcqGGUaGlcqaI5aqoaeaacqaIXaqmcqGGUaGlcqaIWaamaeaacqaIXaqmcqGGUaGlcqaIYaGmaaaaaaqaauaabeqacqaaaaqaaiabigdaXiabc6caUiabikdaYaqaaiabigdaXiabc6caUiabicdaWaqaaiabicdaWiabc6caUiabiIda4aqaaiabigdaXiabc6caUiabiwda1aqaaiabigdaXiabc6caUiabiwda1aqaaiabigdaXiabc6caUiabiEda3aqaaiabigdaXiabc6caUiabiAda2aqaaiabicdaWiabc6caUiabikdaYaaaaeaafaqabeWaeaaaaeaacqaIXaqmcqGGUaGlcqaI3aWnaeaacqaIYaGmcqGGUaGlcqaIXaqmaeaacqaIYaGmcqGGUaGlcqaIZaWmaeaacqaIXaqmcqGGUaGlcqaI4aaoaeaacqaIYaGmcqGGUaGlcqaIWaamaeaacqaIYaGmcqGGUaGlcqaIWaamaeaacqaIXaqmcqGGUaGlcqaI5aqoaeaacqaIWaamcqGGUaGlcqaIZaWmaeaacqaIXaqmcqGGUaGlcqaI2aGnaeaacqaIXaqmcqGGUaGlcqaIYaGmaeaacqaIXaqmcqGGUaGlcqaI0aanaeaacqaIXaqmcqGGUaGlcqaIYaGmaaaaaaGaayjkaiaawMcaaiabc6caUaaa@BEC2@

Then the centroid matrix A¯3×4
 MathType@MTEF@5@5@+=feaafiart1ev1aaatCvAUfKttLearuWrP9MDH5MBPbIqV92AaeXatLxBI9gBaebbnrfifHhDYfgasaacH8akY=wiFfYdH8Gipec8Eeeu0xXdbba9frFj0=OqFfea0dXdd9vqai=hGuQ8kuc9pgc9s8qqaq=dirpe0xb9q8qiLsFr0=vr0=vr0dc8meaabaqaciaacaGaaeqabaqabeGadaaakeaacuWGbbqqgaqeamaaBaaaleaacqaIZaWmcqGHxdaTcqaI0aanaeqaaaaa@31FC@ is

A¯3×4=(0.80.80.81.01.21.21.21.01.61.61.61.0),
 MathType@MTEF@5@5@+=feaafiart1ev1aaatCvAUfKttLearuWrP9MDH5MBPbIqV92AaeXatLxBI9gBaebbnrfifHhDYfgasaacH8akY=wiFfYdH8Gipec8Eeeu0xXdbba9frFj0=OqFfea0dXdd9vqai=hGuQ8kuc9pgc9s8qqaq=dirpe0xb9q8qiLsFr0=vr0=vr0dc8meaabaqaciaacaGaaeqabaqabeGadaaakeaacuWGbbqqgaqeamaaBaaaleaacqaIZaWmcqGHxdaTcqaI0aanaeqaaOGaeyypa0ZaaeWaaeaafaqabeWaeaaaaeaacqaIWaamcqGGUaGlcqaI4aaoaeaacqaIWaamcqGGUaGlcqaI4aaoaeaacqaIWaamcqGGUaGlcqaI4aaoaeaacqaIXaqmcqGGUaGlcqaIWaamaeaacqaIXaqmcqGGUaGlcqaIYaGmaeaacqaIXaqmcqGGUaGlcqaIYaGmaeaacqaIXaqmcqGGUaGlcqaIYaGmaeaacqaIXaqmcqGGUaGlcqaIWaamaeaacqaIXaqmcqGGUaGlcqaI2aGnaeaacqaIXaqmcqGGUaGlcqaI2aGnaeaacqaIXaqmcqGGUaGlcqaI2aGnaeaacqaIXaqmcqGGUaGlcqaIWaamaaaacaGLOaGaayzkaaGaeiilaWcaaa@5702@

and the mean of the centroids is

A^
 MathType@MTEF@5@5@+=feaafiart1ev1aaatCvAUfKttLearuWrP9MDH5MBPbIqV92AaeXatLxBI9gBaebbnrfifHhDYfgasaacH8akY=wiFfYdH8Gipec8Eeeu0xXdbba9frFj0=OqFfea0dXdd9vqai=hGuQ8kuc9pgc9s8qqaq=dirpe0xb9q8qiLsFr0=vr0=vr0dc8meaabaqaciaacaGaaeqabaqabeGadaaakeaacuWGbbqqgaqcaaaa@2DC7@ = (1.2, 1.2, 1.2, 1.0).

The deviation matrix *X*_12×4 _is

X12×4=(0.150.60.60.30.050.20.60.00.10.40.00.3¯0.30.50.60.50.10.20.80.30.30.60.00.60.10.30.20.2¯0.40.60.80.50.10.10.00.80.10.50.70.80.40.40.30.70.00.40.20.2),
 MathType@MTEF@5@5@+=feaafiart1ev1aaatCvAUfKttLearuWrP9MDH5MBPbIqV92AaeXatLxBI9gBaebbnrfifHhDYfgasaacH8akY=wiFfYdH8Gipec8Eeeu0xXdbba9frFj0=OqFfea0dXdd9vqai=hGuQ8kuc9pgc9s8qqaq=dirpe0xb9q8qiLsFr0=vr0=vr0dc8meaabaqaciaacaGaaeqabaqabeGadaaakeaacqWGybawdaWgaaWcbaGaeGymaeJaeGOmaiJaey41aqRaeGinaqdabeaakiabg2da9maabmaabaqbaeGabqqaaaaabaWaaWaaaeaafaqaceWaeaaaaeaacqaIWaamcqGGUaGlcqaIXaqmcqaI1aqnaeaacqaIWaamcqGGUaGlcqaI2aGnaeaacqaIWaamcqGGUaGlcqaI2aGnaeaacqaIWaamcqGGUaGlcqaIZaWmaeaacqaIWaamcqGGUaGlcqaIWaamcqaI1aqnaeaacqaIWaamcqGGUaGlcqaIYaGmaeaacqaIWaamcqGGUaGlcqaI2aGnaeaacqaIWaamcqGGUaGlcqaIWaamaeaacqaIWaamcqGGUaGlcqaIXaqmaeaacqaIWaamcqGGUaGlcqaI0aanaeaacqaIWaamcqGGUaGlcqaIWaamaeaacqaIWaamcqGGUaGlcqaIZaWmaaaaaaqaamaamaaabaqbaeaabqabaaaaaeaacqaIWaamcqGGUaGlcqaIZaWmaeaacqaIWaamcqGGUaGlcqaI1aqnaeaacqaIWaamcqGGUaGlcqaI2aGnaeaacqaIWaamcqGGUaGlcqaI1aqnaeaacqaIWaamcqGGUaGlcqaIXaqmaeaacqaIWaamcqGGUaGlcqaIYaGmaeaacqaIWaamcqGGUaGlcqaI4aaoaeaacqaIWaamcqGGUaGlcqaIZaWmaeaacqaIWaamcqGGUaGlcqaIZaWmaeaacqaIWaamcqGGUaGlcqaI2aGnaeaacqaIWaamcqGGUaGlcqaIWaamaeaacqaIWaamcqGGUaGlcqaI2aGnaeaacqaIWaamcqGGUaGlcqaIXaqmaeaacqaIWaamcqGGUaGlcqaIZaWmaeaacqaIWaamcqGGUaGlcqaIYaGmaeaacqaIWaamcqGGUaGlcqaIYaGmaaaaaaqaauaabeqacqaaaaqaaiabicdaWiabc6caUiabisda0aqaaiabicdaWiabc6caUiabiAda2aqaaiabicdaWiabc6caUiabiIda4aqaaiabicdaWiabc6caUiabiwda1aqaaiabicdaWiabc6caUiabigdaXaqaaiabicdaWiabc6caUiabigdaXaqaaiabicdaWiabc6caUiabicdaWaqaaiabicdaWiabc6caUiabiIda4aaaaeaafaqabeWaeaaaaeaacqaIWaamcqGGUaGlcqaIXaqmaeaacqaIWaamcqGGUaGlcqaI1aqnaeaacqaIWaamcqGGUaGlcqaI3aWnaeaacqaIWaamcqGGUaGlcqaI4aaoaeaacqaIWaamcqGGUaGlcqaI0aanaeaacqaIWaamcqGGUaGlcqaI0aanaeaacqaIWaamcqGGUaGlcqaIZaWmaeaacqaIWaamcqGGUaGlcqaI3aWnaeaacqaIWaamcqGGUaGlcqaIWaamaeaacqaIWaamcqGGUaGlcqaI0aanaeaacqaIWaamcqGGUaGlcqaIYaGmaeaacqaIWaamcqGGUaGlcqaIYaGmaaaaaaGaayjkaiaawMcaaiabcYcaSaaa@BE57@

and the intra-class average deviations are

X¯3×4=(0.10.40.40.20.20.40.40.40.20.40.40.6).
 MathType@MTEF@5@5@+=feaafiart1ev1aaatCvAUfKttLearuWrP9MDH5MBPbIqV92AaeXatLxBI9gBaebbnrfifHhDYfgasaacH8akY=wiFfYdH8Gipec8Eeeu0xXdbba9frFj0=OqFfea0dXdd9vqai=hGuQ8kuc9pgc9s8qqaq=dirpe0xb9q8qiLsFr0=vr0=vr0dc8meaabaqaciaacaGaaeqabaqabeGadaaakeaacuWGybawgaqeamaaBaaaleaacqaIZaWmcqGHxdaTcqaI0aanaeqaaOGaeyypa0ZaaeWaaeaafaqabeWaeaaaaeaacqaIWaamcqGGUaGlcqaIXaqmaeaacqaIWaamcqGGUaGlcqaI0aanaeaacqaIWaamcqGGUaGlcqaI0aanaeaacqaIWaamcqGGUaGlcqaIYaGmaeaacqaIWaamcqGGUaGlcqaIYaGmaeaacqaIWaamcqGGUaGlcqaI0aanaeaacqaIWaamcqGGUaGlcqaI0aanaeaacqaIWaamcqGGUaGlcqaI0aanaeaacqaIWaamcqGGUaGlcqaIYaGmaeaacqaIWaamcqGGUaGlcqaI0aanaeaacqaIWaamcqGGUaGlcqaI0aanaeaacqaIWaamcqGGUaGlcqaI2aGnaaaacaGLOaGaayzkaaGaeiOla4caaa@5714@

**Figure 3:**
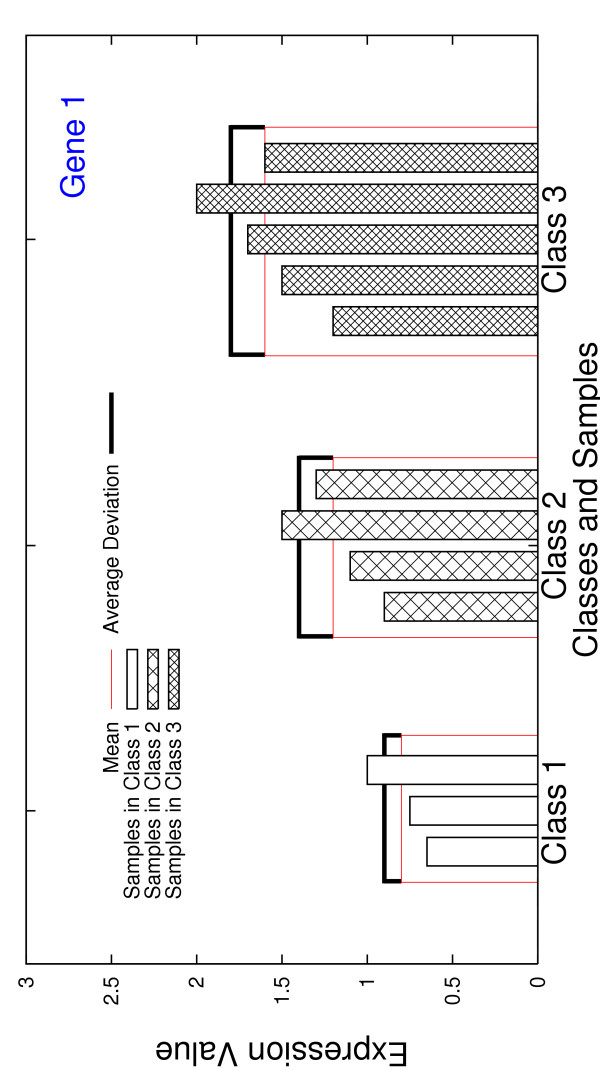
The plot of the expression values of gene 1 across all 12 samples in the example dataset, with both intra-class means and average deviations calculated.

**Figure 4:**
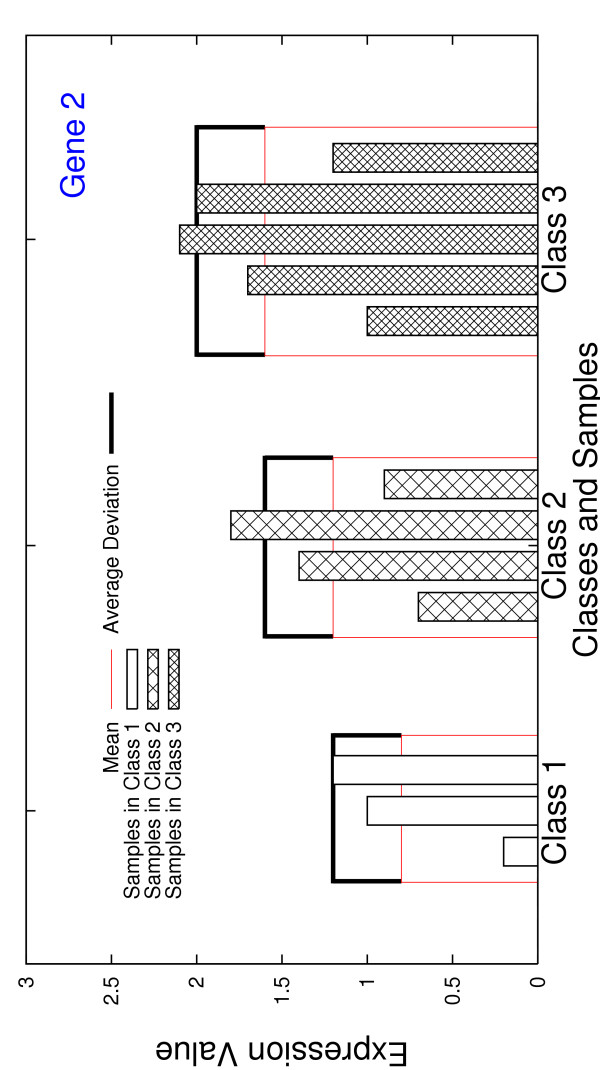
The plot of the expression values of gene 2 across all 12 samples in the example dataset, with both intra-class means and average deviations calculated.

**Figure 5:**
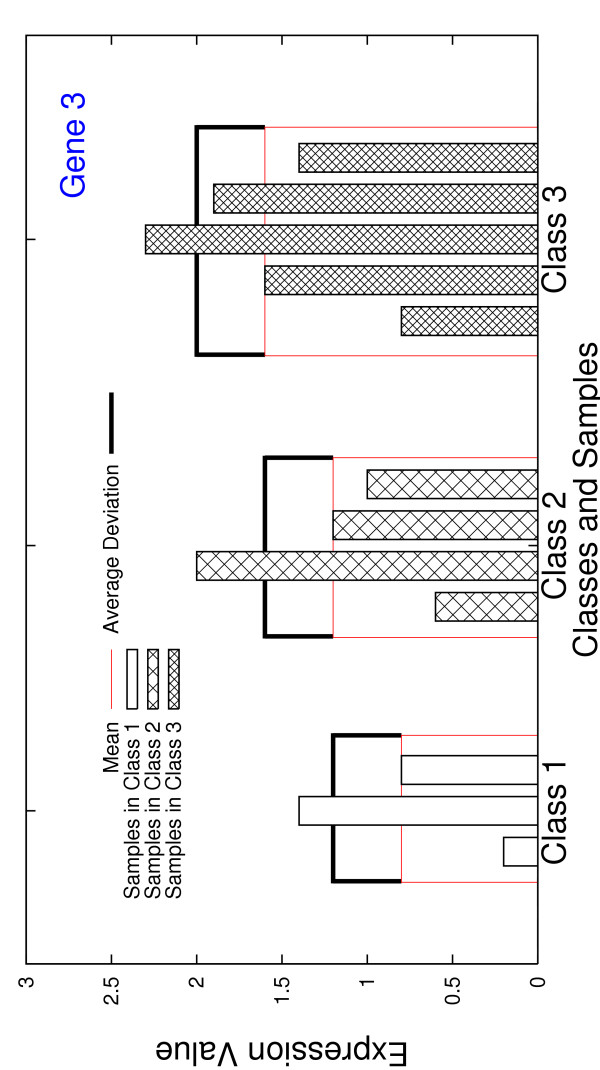
The plot of the expression values of gene 3 across all 12 samples in the example dataset, with both intra-class means and average deviations calculated.

**Figure 6:**
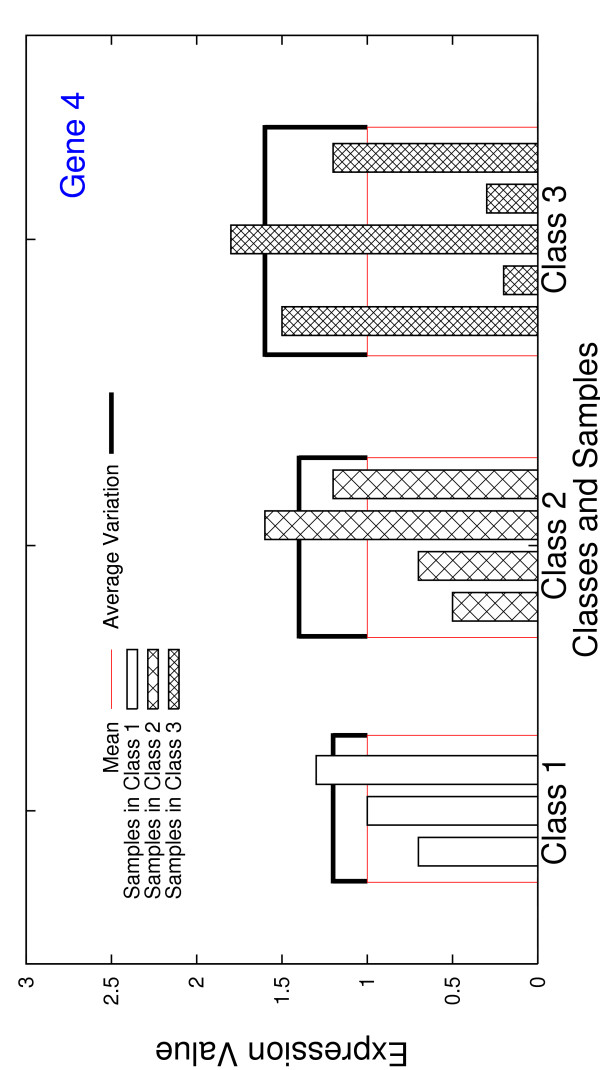
The plot of the expression values of gene 4 across all 12 samples in the example dataset, with both intra-class means and average deviations calculated.

Figures 3, 4, 5, 6 illustrate the expression values of these four genes across all 12 samples, with the intra-class means and average deviations also shown. There are three key ideas in our design of gene scoring functions, which will be exemplified through these four genes. First of all, gene 1 has quite different mean expression values across three classes, compared to gene 4 that has the same means. Therefore, gene 1 is intuitively better than gene 4 in terms of discriminating power. Note that the goal of gene selection is to select genes that have significantly different means across different classes. For each gene *j*, the quantity a^j
 MathType@MTEF@5@5@+=feaafiart1ev1aaatCvAUfKttLearuWrP9MDH5MBPbIqV92AaeXatLxBI9gBaebbnrfifHhDYfgasaacH8akY=wiFfYdH8Gipec8Eeeu0xXdbba9frFj0=OqFfea0dXdd9vqai=hGuQ8kuc9pgc9s8qqaq=dirpe0xb9q8qiLsFr0=vr0=vr0dc8meaabaqaciaacaGaaeqabaqabeGadaaakeaacuWGHbqygaqcamaaBaaaleaacqWGQbGAaeqaaaaa@2F90@ is the mean of all the centroids on gene *j *and it represents all the samples. a^j
 MathType@MTEF@5@5@+=feaafiart1ev1aaatCvAUfKttLearuWrP9MDH5MBPbIqV92AaeXatLxBI9gBaebbnrfifHhDYfgasaacH8akY=wiFfYdH8Gipec8Eeeu0xXdbba9frFj0=OqFfea0dXdd9vqai=hGuQ8kuc9pgc9s8qqaq=dirpe0xb9q8qiLsFr0=vr0=vr0dc8meaabaqaciaacaGaaeqabaqabeGadaaakeaacuWGHbqygaqcamaaBaaaleaacqWGQbGAaeqaaaaa@2F90@ is stable, that is, it would not change when the samples in one class are duplicated (since the number of classes, *L*, and all the means, a¯kj
 MathType@MTEF@5@5@+=feaafiart1ev1aaatCvAUfKttLearuWrP9MDH5MBPbIqV92AaeXatLxBI9gBaebbnrfifHhDYfgasaacH8akY=wiFfYdH8Gipec8Eeeu0xXdbba9frFj0=OqFfea0dXdd9vqai=hGuQ8kuc9pgc9s8qqaq=dirpe0xb9q8qiLsFr0=vr0=vr0dc8meaabaqaciaacaGaaeqabaqabeGadaaakeaacuWGHbqygaqeamaaBaaaleaacqWGRbWAcqWGQbGAaeqaaaaa@30F7@, for *k *= 1, 2,..., *L*, do not change). We define the *scatter *of gene *j *to capture the *inter-class *variations, which takes in a^j
 MathType@MTEF@5@5@+=feaafiart1ev1aaatCvAUfKttLearuWrP9MDH5MBPbIqV92AaeXatLxBI9gBaebbnrfifHhDYfgasaacH8akY=wiFfYdH8Gipec8Eeeu0xXdbba9frFj0=OqFfea0dXdd9vqai=hGuQ8kuc9pgc9s8qqaq=dirpe0xb9q8qiLsFr0=vr0=vr0dc8meaabaqaciaacaGaaeqabaqabeGadaaakeaacuWGHbqygaqcamaaBaaaleaacqWGQbGAaeqaaaaa@2F90@ as a component:

scatter(j)=1L∑k=1L(a¯kj−a^j)2+12min⁡w≠v|a¯wj−a^vj|,
 MathType@MTEF@5@5@+=feaafiart1ev1aaatCvAUfKttLearuWrP9MDH5MBPbIqV92AaeXatLxBI9gBaebbnrfifHhDYfgasaacH8akY=wiFfYdH8Gipec8Eeeu0xXdbba9frFj0=OqFfea0dXdd9vqai=hGuQ8kuc9pgc9s8qqaq=dirpe0xb9q8qiLsFr0=vr0=vr0dc8meaabaqaciaacaGaaeqabaqabeGadaaakeaacqWGZbWCcqWGJbWycqWGHbqycqWG0baDcqWG0baDcqWGLbqzcqWGYbGCcqGGOaakcqWGQbGAcqGGPaqkcqGH9aqpdaGcaaqaamaalaaabaGaeGymaedabaGaemitaWeaamaaqahabaGaeiikaGIafmyyaeMbaebadaWgaaWcbaGaem4AaSMaemOAaOgabeaakiabgkHiTiqbdggaHzaajaWaaSbaaSqaaiabdQgaQbqabaGccqGGPaqkdaahaaWcbeqaaiabikdaYaaaaeaacqWGRbWAcqGH9aqpcqaIXaqmaeaacqWGmbata0GaeyyeIuoaaSqabaGccqGHRaWkdaWcaaqaaiabigdaXaqaaiabikdaYaaadaWfqaqaaiGbc2gaTjabcMgaPjabc6gaUbWcbaGaem4DaCNaeyiyIKRaemODayhabeaakmaaemaabaGafmyyaeMbaebadaWgaaWcbaGaem4DaCNaemOAaOgabeaakiabgkHiTiqbdggaHzaajaWaaSbaaSqaaiabdAha2jabdQgaQbqabaaakiaawEa7caGLiWoacqGGSaalaaa@680B@

in which the square root is the standard (estimated) deviation of all the centroids on gene *j*. Clearly seen, *scatter*(*j*) is a stable function. More discriminatory genes are expected to have bigger *scatter*-values. In the following, we prove an upper bound and a lower bound for *scatter*(*j*).

**Lemma 1 ***Given n arbitrary nonnegative numbers a*_1_, *a*_2_,...,*a*_*n*_, *the inequality *1n
 MathType@MTEF@5@5@+=feaafiart1ev1aaatCvAUfKttLearuWrP9MDH5MBPbIqV92AaeXatLxBI9gBaebbnrfifHhDYfgasaacH8akY=wiFfYdH8Gipec8Eeeu0xXdbba9frFj0=OqFfea0dXdd9vqai=hGuQ8kuc9pgc9s8qqaq=dirpe0xb9q8qiLsFr0=vr0=vr0dc8meaabaqaciaacaGaaeqabaqabeGadaaakeaadaWcaaqaaiabigdaXaqaaiabd6gaUbaaaaa@2F11@(*a*_1 _+ *a*_2 _+ ... + *a*_*n*_) ≤ 1n(a12+a22+…+an2)
 MathType@MTEF@5@5@+=feaafiart1ev1aaatCvAUfKttLearuWrP9MDH5MBPbIqV92AaeXatLxBI9gBaebbnrfifHhDYfgasaacH8akY=wiFfYdH8Gipec8Eeeu0xXdbba9frFj0=OqFfea0dXdd9vqai=hGuQ8kuc9pgc9s8qqaq=dirpe0xb9q8qiLsFr0=vr0=vr0dc8meaabaqaciaacaGaaeqabaqabeGadaaakeaadaGcaaqaamaalaaabaGaeGymaedabaGaemOBa4gaaiabcIcaOiabdggaHnaaDaaaleaacqaIXaqmaeaacqaIYaGmaaGccqGHRaWkcqWGHbqydaqhaaWcbaGaeGOmaidabaGaeGOmaidaaOGaey4kaSIaeSOjGSKaey4kaSIaemyyae2aa0baaSqaaiabd6gaUbqaaiabikdaYaaakiabcMcaPaWcbeaaaaa@3F49@*holds, and it becomes equality if and only if a*_1 _= *a*_2 _= ... = *a*_*n*_.

Lemma 1 can be proven by a mathematical induction.

**Lemma 2 ***Given n arbitrary nonnegative numbers sorted in order a*_1 _≤ *a*_2 _≤ ... ≤ *a*_*n*_, *define *a^=1n∑i=1nai
 MathType@MTEF@5@5@+=feaafiart1ev1aaatCvAUfKttLearuWrP9MDH5MBPbIqV92AaeXatLxBI9gBaebbnrfifHhDYfgasaacH8akY=wiFfYdH8Gipec8Eeeu0xXdbba9frFj0=OqFfea0dXdd9vqai=hGuQ8kuc9pgc9s8qqaq=dirpe0xb9q8qiLsFr0=vr0=vr0dc8meaabaqaciaacaGaaeqabaqabeGadaaakeaacuWGHbqygaqcaiabg2da9maalaaabaGaeGymaedabaGaemOBa4gaamaaqadabaGaemyyae2aaSbaaSqaaiabdMgaPbqabaaabaGaemyAaKMaeyypa0JaeGymaedabaGaemOBa4ganiabggHiLdaaaa@3AF1@, a˜=12
 MathType@MTEF@5@5@+=feaafiart1ev1aaatCvAUfKttLearuWrP9MDH5MBPbIqV92AaeXatLxBI9gBaebbnrfifHhDYfgasaacH8akY=wiFfYdH8Gipec8Eeeu0xXdbba9frFj0=OqFfea0dXdd9vqai=hGuQ8kuc9pgc9s8qqaq=dirpe0xb9q8qiLsFr0=vr0=vr0dc8meaabaqaciaacaGaaeqabaqabeGadaaakeaacuWGHbqygaacaiabg2da9maalaaabaGaeGymaedabaGaeGOmaidaaaaa@30FE@ min_1≤*i*≤*n*-1_(*a*_*i*+1 _- *a*_*i*_), *and *S=1n∑i=1n(ai−a^)2
 MathType@MTEF@5@5@+=feaafiart1ev1aaatCvAUfKttLearuWrP9MDH5MBPbIqV92AaeXatLxBI9gBaebbnrfifHhDYfgasaacH8akY=wiFfYdH8Gipec8Eeeu0xXdbba9frFj0=OqFfea0dXdd9vqai=hGuQ8kuc9pgc9s8qqaq=dirpe0xb9q8qiLsFr0=vr0=vr0dc8meaabaqaciaacaGaaeqabaqabeGadaaakeaacqWGtbWucqGH9aqpdaGcaaqaamaalaaabaGaeGymaedabaGaemOBa4gaamaaqadabaGaeiikaGIaemyyae2aaSbaaSqaaiabdMgaPbqabaGccqGHsislcuWGHbqygaqcaiabcMcaPmaaCaaaleqabaGaeGOmaidaaaqaaiabdMgaPjabg2da9iabigdaXaqaaiabd6gaUbqdcqGHris5aaWcbeaaaaa@4003@*. Then*,

a˜
 MathType@MTEF@5@5@+=feaafiart1ev1aaatCvAUfKttLearuWrP9MDH5MBPbIqV92AaeXatLxBI9gBaebbnrfifHhDYfgasaacH8akY=wiFfYdH8Gipec8Eeeu0xXdbba9frFj0=OqFfea0dXdd9vqai=hGuQ8kuc9pgc9s8qqaq=dirpe0xb9q8qiLsFr0=vr0=vr0dc8meaabaqaciaacaGaaeqabaqabeGadaaakeaacuWGHbqygaacaaaa@2E06@ ≤ *S *≤ (*a*_*n *_- *a*_1_) - a˜
 MathType@MTEF@5@5@+=feaafiart1ev1aaatCvAUfKttLearuWrP9MDH5MBPbIqV92AaeXatLxBI9gBaebbnrfifHhDYfgasaacH8akY=wiFfYdH8Gipec8Eeeu0xXdbba9frFj0=OqFfea0dXdd9vqai=hGuQ8kuc9pgc9s8qqaq=dirpe0xb9q8qiLsFr0=vr0=vr0dc8meaabaqaciaacaGaaeqabaqabeGadaaakeaacuWGHbqygaacaaaa@2E06@.

PROOF. Note that both *S *and a˜
 MathType@MTEF@5@5@+=feaafiart1ev1aaatCvAUfKttLearuWrP9MDH5MBPbIqV92AaeXatLxBI9gBaebbnrfifHhDYfgasaacH8akY=wiFfYdH8Gipec8Eeeu0xXdbba9frFj0=OqFfea0dXdd9vqai=hGuQ8kuc9pgc9s8qqaq=dirpe0xb9q8qiLsFr0=vr0=vr0dc8meaabaqaciaacaGaaeqabaqabeGadaaakeaacuWGHbqygaacaaaa@2E06@ are nonnegative. Therefore, if a˜
 MathType@MTEF@5@5@+=feaafiart1ev1aaatCvAUfKttLearuWrP9MDH5MBPbIqV92AaeXatLxBI9gBaebbnrfifHhDYfgasaacH8akY=wiFfYdH8Gipec8Eeeu0xXdbba9frFj0=OqFfea0dXdd9vqai=hGuQ8kuc9pgc9s8qqaq=dirpe0xb9q8qiLsFr0=vr0=vr0dc8meaabaqaciaacaGaaeqabaqabeGadaaakeaacuWGHbqygaacaaaa@2E06@ = 0, then *S *≥ a˜
 MathType@MTEF@5@5@+=feaafiart1ev1aaatCvAUfKttLearuWrP9MDH5MBPbIqV92AaeXatLxBI9gBaebbnrfifHhDYfgasaacH8akY=wiFfYdH8Gipec8Eeeu0xXdbba9frFj0=OqFfea0dXdd9vqai=hGuQ8kuc9pgc9s8qqaq=dirpe0xb9q8qiLsFr0=vr0=vr0dc8meaabaqaciaacaGaaeqabaqabeGadaaakeaacuWGHbqygaacaaaa@2E06@ holds trivially. In the other case, we have *a*_1 _<*a*_2 _< ... <*a*_*n*_. Assume without loss of generality that the minimum is achieved at *i *= *k*, that is, a˜=12
 MathType@MTEF@5@5@+=feaafiart1ev1aaatCvAUfKttLearuWrP9MDH5MBPbIqV92AaeXatLxBI9gBaebbnrfifHhDYfgasaacH8akY=wiFfYdH8Gipec8Eeeu0xXdbba9frFj0=OqFfea0dXdd9vqai=hGuQ8kuc9pgc9s8qqaq=dirpe0xb9q8qiLsFr0=vr0=vr0dc8meaabaqaciaacaGaaeqabaqabeGadaaakeaacuWGHbqygaacaiabg2da9maalaaabaGaeGymaedabaGaeGOmaidaaaaa@30FE@ (*a*_*k*+1 _- *a*_*k*_). If a^
 MathType@MTEF@5@5@+=feaafiart1ev1aaatCvAUfKttLearuWrP9MDH5MBPbIqV92AaeXatLxBI9gBaebbnrfifHhDYfgasaacH8akY=wiFfYdH8Gipec8Eeeu0xXdbba9frFj0=OqFfea0dXdd9vqai=hGuQ8kuc9pgc9s8qqaq=dirpe0xb9q8qiLsFr0=vr0=vr0dc8meaabaqaciaacaGaaeqabaqabeGadaaakeaacuWGHbqygaqcaaaa@2E07@ ∈ [*a*_*k*_, *a*_*k*+1_], from Lemma 1, we have

12((ak−a^)2+(ak+1−a^)2)≥(12((a^−ak)+(ak+1−a^))2=a˜2.
 MathType@MTEF@5@5@+=feaafiart1ev1aaatCvAUfKttLearuWrP9MDH5MBPbIqV92AaeXatLxBI9gBaebbnrfifHhDYfgasaacH8akY=wiFfYdH8Gipec8Eeeu0xXdbba9frFj0=OqFfea0dXdd9vqai=hGuQ8kuc9pgc9s8qqaq=dirpe0xb9q8qiLsFr0=vr0=vr0dc8meaabaqaciaacaGaaeqabaqabeGadaaakeaadaWcaaqaaiabigdaXaqaaiabikdaYaaadaqadaqaaiabcIcaOiabdggaHnaaBaaaleaacqWGRbWAaeqaaOGaeyOeI0IafmyyaeMbaKaacqGGPaqkdaahaaWcbeqaaiabikdaYaaakiabgUcaRiabcIcaOiabdggaHnaaBaaaleaacqWGRbWAcqGHRaWkcqaIXaqmaeqaaOGaeyOeI0IafmyyaeMbaKaacqGGPaqkdaahaaWcbeqaaiabikdaYaaaaOGaayjkaiaawMcaaiabgwMiZoaabmaabaWaaSaaaeaacqaIXaqmaeaacqaIYaGmaaGaeiikaGIaeiikaGIafmyyaeMbaKaacqGHsislcqWGHbqydaWgaaWcbaGaem4AaSgabeaakiabcMcaPiabgUcaRiabcIcaOiabdggaHnaaBaaaleaacqWGRbWAcqGHRaWkcqaIXaqmaeqaaOGaeyOeI0IafmyyaeMbaKaacqGGPaqkaiaawIcacaGLPaaadaahaaWcbeqaaiabikdaYaaakiabg2da9iqbdggaHzaaiaWaaWbaaSqabeaacqaIYaGmaaGccqGGUaGlaaa@5EF8@

For *i *≠ *k*, *k *+ 1, (*a*_*i *_≥ a^
 MathType@MTEF@5@5@+=feaafiart1ev1aaatCvAUfKttLearuWrP9MDH5MBPbIqV92AaeXatLxBI9gBaebbnrfifHhDYfgasaacH8akY=wiFfYdH8Gipec8Eeeu0xXdbba9frFj0=OqFfea0dXdd9vqai=hGuQ8kuc9pgc9s8qqaq=dirpe0xb9q8qiLsFr0=vr0=vr0dc8meaabaqaciaacaGaaeqabaqabeGadaaakeaacuWGHbqygaqcaaaa@2E07@)^2 ^≥ a˜
 MathType@MTEF@5@5@+=feaafiart1ev1aaatCvAUfKttLearuWrP9MDH5MBPbIqV92AaeXatLxBI9gBaebbnrfifHhDYfgasaacH8akY=wiFfYdH8Gipec8Eeeu0xXdbba9frFj0=OqFfea0dXdd9vqai=hGuQ8kuc9pgc9s8qqaq=dirpe0xb9q8qiLsFr0=vr0=vr0dc8meaabaqaciaacaGaaeqabaqabeGadaaakeaacuWGHbqygaacaaaa@2E06@^2^. Therefore, nS2=∑i=1n(ai−a^)2≥na˜2
 MathType@MTEF@5@5@+=feaafiart1ev1aaatCvAUfKttLearuWrP9MDH5MBPbIqV92AaeXatLxBI9gBaebbnrfifHhDYfgasaacH8akY=wiFfYdH8Gipec8Eeeu0xXdbba9frFj0=OqFfea0dXdd9vqai=hGuQ8kuc9pgc9s8qqaq=dirpe0xb9q8qiLsFr0=vr0=vr0dc8meaabaqaciaacaGaaeqabaqabeGadaaakeaacqWGUbGBcqWGtbWudaahaaWcbeqaaiabikdaYaaakiabg2da9maaqadabaGaeiikaGIaemyyae2aaSbaaSqaaiabdMgaPbqabaGccqGHsislcuWGHbqygaqcaiabcMcaPmaaCaaaleqabaGaeGOmaidaaaqaaiabdMgaPjabg2da9iabigdaXaqaaiabd6gaUbqdcqGHris5aOGaeyyzImRaemOBa4MafmyyaeMbaGaadaahaaWcbeqaaiabikdaYaaaaaa@45BF@. if a^
 MathType@MTEF@5@5@+=feaafiart1ev1aaatCvAUfKttLearuWrP9MDH5MBPbIqV92AaeXatLxBI9gBaebbnrfifHhDYfgasaacH8akY=wiFfYdH8Gipec8Eeeu0xXdbba9frFj0=OqFfea0dXdd9vqai=hGuQ8kuc9pgc9s8qqaq=dirpe0xb9q8qiLsFr0=vr0=vr0dc8meaabaqaciaacaGaaeqabaqabeGadaaakeaacuWGHbqygaqcaaaa@2E07@ ∈ [*a*_*p*_, *a*_*p*+1_] but *p *≠ *k*, similarly we will have 12
 MathType@MTEF@5@5@+=feaafiart1ev1aaatCvAUfKttLearuWrP9MDH5MBPbIqV92AaeXatLxBI9gBaebbnrfifHhDYfgasaacH8akY=wiFfYdH8Gipec8Eeeu0xXdbba9frFj0=OqFfea0dXdd9vqai=hGuQ8kuc9pgc9s8qqaq=dirpe0xb9q8qiLsFr0=vr0=vr0dc8meaabaqaciaacaGaaeqabaqabeGadaaakeaadaWcaaqaaiabigdaXaqaaiabikdaYaaaaaa@2E9E@((*a*_*p *_- a^
 MathType@MTEF@5@5@+=feaafiart1ev1aaatCvAUfKttLearuWrP9MDH5MBPbIqV92AaeXatLxBI9gBaebbnrfifHhDYfgasaacH8akY=wiFfYdH8Gipec8Eeeu0xXdbba9frFj0=OqFfea0dXdd9vqai=hGuQ8kuc9pgc9s8qqaq=dirpe0xb9q8qiLsFr0=vr0=vr0dc8meaabaqaciaacaGaaeqabaqabeGadaaakeaacuWGHbqygaqcaaaa@2E07@)^2 ^+ (*a*_*p*+1 _- a^
 MathType@MTEF@5@5@+=feaafiart1ev1aaatCvAUfKttLearuWrP9MDH5MBPbIqV92AaeXatLxBI9gBaebbnrfifHhDYfgasaacH8akY=wiFfYdH8Gipec8Eeeu0xXdbba9frFj0=OqFfea0dXdd9vqai=hGuQ8kuc9pgc9s8qqaq=dirpe0xb9q8qiLsFr0=vr0=vr0dc8meaabaqaciaacaGaaeqabaqabeGadaaakeaacuWGHbqygaqcaaaa@2E07@)^2^) ≥ (*a*_*p*+1 _- *a*_*p*_)^2 ^≥ a˜
 MathType@MTEF@5@5@+=feaafiart1ev1aaatCvAUfKttLearuWrP9MDH5MBPbIqV92AaeXatLxBI9gBaebbnrfifHhDYfgasaacH8akY=wiFfYdH8Gipec8Eeeu0xXdbba9frFj0=OqFfea0dXdd9vqai=hGuQ8kuc9pgc9s8qqaq=dirpe0xb9q8qiLsFr0=vr0=vr0dc8meaabaqaciaacaGaaeqabaqabeGadaaakeaacuWGHbqygaacaaaa@2E06@^2 ^and for *i *≠ *p*, *p *+ 1, (*a*_*i *_- a^
 MathType@MTEF@5@5@+=feaafiart1ev1aaatCvAUfKttLearuWrP9MDH5MBPbIqV92AaeXatLxBI9gBaebbnrfifHhDYfgasaacH8akY=wiFfYdH8Gipec8Eeeu0xXdbba9frFj0=OqFfea0dXdd9vqai=hGuQ8kuc9pgc9s8qqaq=dirpe0xb9q8qiLsFr0=vr0=vr0dc8meaabaqaciaacaGaaeqabaqabeGadaaakeaacuWGHbqygaqcaaaa@2E07@)^2 ^≥ a˜
 MathType@MTEF@5@5@+=feaafiart1ev1aaatCvAUfKttLearuWrP9MDH5MBPbIqV92AaeXatLxBI9gBaebbnrfifHhDYfgasaacH8akY=wiFfYdH8Gipec8Eeeu0xXdbba9frFj0=OqFfea0dXdd9vqai=hGuQ8kuc9pgc9s8qqaq=dirpe0xb9q8qiLsFr0=vr0=vr0dc8meaabaqaciaacaGaaeqabaqabeGadaaakeaacuWGHbqygaacaaaa@2E06@^2^. This proves that a˜
 MathType@MTEF@5@5@+=feaafiart1ev1aaatCvAUfKttLearuWrP9MDH5MBPbIqV92AaeXatLxBI9gBaebbnrfifHhDYfgasaacH8akY=wiFfYdH8Gipec8Eeeu0xXdbba9frFj0=OqFfea0dXdd9vqai=hGuQ8kuc9pgc9s8qqaq=dirpe0xb9q8qiLsFr0=vr0=vr0dc8meaabaqaciaacaGaaeqabaqabeGadaaakeaacuWGHbqygaacaaaa@2E06@ ≤ *S*.

Inequality *S *+ a˜
 MathType@MTEF@5@5@+=feaafiart1ev1aaatCvAUfKttLearuWrP9MDH5MBPbIqV92AaeXatLxBI9gBaebbnrfifHhDYfgasaacH8akY=wiFfYdH8Gipec8Eeeu0xXdbba9frFj0=OqFfea0dXdd9vqai=hGuQ8kuc9pgc9s8qqaq=dirpe0xb9q8qiLsFr0=vr0=vr0dc8meaabaqaciaacaGaaeqabaqabeGadaaakeaacuWGHbqygaacaaaa@2E06@ ≤ *a*_*n *_- *a*_1 _holds again if a˜
 MathType@MTEF@5@5@+=feaafiart1ev1aaatCvAUfKttLearuWrP9MDH5MBPbIqV92AaeXatLxBI9gBaebbnrfifHhDYfgasaacH8akY=wiFfYdH8Gipec8Eeeu0xXdbba9frFj0=OqFfea0dXdd9vqai=hGuQ8kuc9pgc9s8qqaq=dirpe0xb9q8qiLsFr0=vr0=vr0dc8meaabaqaciaacaGaaeqabaqabeGadaaakeaacuWGHbqygaacaaaa@2E06@ = 0, since (*a*_*i *_- a^
 MathType@MTEF@5@5@+=feaafiart1ev1aaatCvAUfKttLearuWrP9MDH5MBPbIqV92AaeXatLxBI9gBaebbnrfifHhDYfgasaacH8akY=wiFfYdH8Gipec8Eeeu0xXdbba9frFj0=OqFfea0dXdd9vqai=hGuQ8kuc9pgc9s8qqaq=dirpe0xb9q8qiLsFr0=vr0=vr0dc8meaabaqaciaacaGaaeqabaqabeGadaaakeaacuWGHbqygaqcaaaa@2E07@)^2 ^≤ (*a*_*n *_- *a*_1_)^2 ^for every *i*. Therefore, we may assume that *a*_1 _<*a*_2 _< ... <*a*_*n*_. A similar enlarging process gives *S *≤ max{*a*_*n *_- a^
 MathType@MTEF@5@5@+=feaafiart1ev1aaatCvAUfKttLearuWrP9MDH5MBPbIqV92AaeXatLxBI9gBaebbnrfifHhDYfgasaacH8akY=wiFfYdH8Gipec8Eeeu0xXdbba9frFj0=OqFfea0dXdd9vqai=hGuQ8kuc9pgc9s8qqaq=dirpe0xb9q8qiLsFr0=vr0=vr0dc8meaabaqaciaacaGaaeqabaqabeGadaaakeaacuWGHbqygaqcaaaa@2E07@, a^
 MathType@MTEF@5@5@+=feaafiart1ev1aaatCvAUfKttLearuWrP9MDH5MBPbIqV92AaeXatLxBI9gBaebbnrfifHhDYfgasaacH8akY=wiFfYdH8Gipec8Eeeu0xXdbba9frFj0=OqFfea0dXdd9vqai=hGuQ8kuc9pgc9s8qqaq=dirpe0xb9q8qiLsFr0=vr0=vr0dc8meaabaqaciaacaGaaeqabaqabeGadaaakeaacuWGHbqygaqcaaaa@2E07@ - *a*_1_}. Since

an−a^+a˜≤an−12(a2+a1)+12(a2−a1)=an−a1
 MathType@MTEF@5@5@+=feaafiart1ev1aaatCvAUfKttLearuWrP9MDH5MBPbIqV92AaeXatLxBI9gBaebbnrfifHhDYfgasaacH8akY=wiFfYdH8Gipec8Eeeu0xXdbba9frFj0=OqFfea0dXdd9vqai=hGuQ8kuc9pgc9s8qqaq=dirpe0xb9q8qiLsFr0=vr0=vr0dc8meaabaqaciaacaGaaeqabaqabeGadaaakeaacqWGHbqydaWgaaWcbaGaemOBa4gabeaakiabgkHiTiqbdggaHzaajaGaey4kaSIafmyyaeMbaGaacqGHKjYOcqWGHbqydaWgaaWcbaGaemOBa4gabeaakiabgkHiTmaalaaabaGaeGymaedabaGaeGOmaidaaiabcIcaOiabdggaHnaaBaaaleaacqaIYaGmaeqaaOGaey4kaSIaemyyae2aaSbaaSqaaiabigdaXaqabaGccqGGPaqkcqGHRaWkdaWcaaqaaiabigdaXaqaaiabikdaYaaacqGGOaakcqWGHbqydaWgaaWcbaGaeGOmaidabeaakiabgkHiTiabdggaHnaaBaaaleaacqaIXaqmaeqaaOGaeiykaKIaeyypa0Jaemyyae2aaSbaaSqaaiabd6gaUbqabaGccqGHsislcqWGHbqydaWgaaWcbaGaeGymaedabeaaaaa@549F@

and

a^−a1+a˜≤12(an+an−1)−a1+12(an−an−1)=an−a1,
 MathType@MTEF@5@5@+=feaafiart1ev1aaatCvAUfKttLearuWrP9MDH5MBPbIqV92AaeXatLxBI9gBaebbnrfifHhDYfgasaacH8akY=wiFfYdH8Gipec8Eeeu0xXdbba9frFj0=OqFfea0dXdd9vqai=hGuQ8kuc9pgc9s8qqaq=dirpe0xb9q8qiLsFr0=vr0=vr0dc8meaabaqaciaacaGaaeqabaqabeGadaaakeaacuWGHbqygaqcaiabgkHiTiabdggaHnaaBaaaleaacqaIXaqmaeqaaOGaey4kaSIafmyyaeMbaGaacqGHKjYOdaWcaaqaaiabigdaXaqaaiabikdaYaaacqGGOaakcqWGHbqydaWgaaWcbaGaemOBa4gabeaakiabgUcaRiabdggaHnaaBaaaleaacqWGUbGBcqGHsislcqaIXaqmaeqaaOGaeiykaKIaeyOeI0Iaemyyae2aaSbaaSqaaiabigdaXaqabaGccqGHRaWkdaWcaaqaaiabigdaXaqaaiabikdaYaaacqGGOaakcqWGHbqydaWgaaWcbaGaemOBa4gabeaakiabgkHiTiabdggaHnaaBaaaleaacqWGUbGBcqGHsislcqaIXaqmaeqaaOGaeiykaKIaeyypa0Jaemyyae2aaSbaaSqaaiabd6gaUbqabaGccqGHsislcqWGHbqydaWgaaWcbaGaeGymaedabeaakiabcYcaSaaa@5A29@

we conclude that *S *+ a˜
 MathType@MTEF@5@5@+=feaafiart1ev1aaatCvAUfKttLearuWrP9MDH5MBPbIqV92AaeXatLxBI9gBaebbnrfifHhDYfgasaacH8akY=wiFfYdH8Gipec8Eeeu0xXdbba9frFj0=OqFfea0dXdd9vqai=hGuQ8kuc9pgc9s8qqaq=dirpe0xb9q8qiLsFr0=vr0=vr0dc8meaabaqaciaacaGaaeqabaqabeGadaaakeaacuWGHbqygaacaaaa@2E06@ ≤ *a*_*n *_- *a*_1_.     □

According to Lemma 2, the following theorem on the bounds on *scatter*(*j*) holds.

**Theorem 3 ***For gene j, define *max(*j*) = max_*w*≠*v *_|a¯wj−a¯vj|
 MathType@MTEF@5@5@+=feaafiart1ev1aaatCvAUfKttLearuWrP9MDH5MBPbIqV92AaeXatLxBI9gBaebbnrfifHhDYfgasaacH8akY=wiFfYdH8Gipec8Eeeu0xXdbba9frFj0=OqFfea0dXdd9vqai=hGuQ8kuc9pgc9s8qqaq=dirpe0xb9q8qiLsFr0=vr0=vr0dc8meaabaqaciaacaGaaeqabaqabeGadaaakeaadaabdaqaaiqbdggaHzaaraWaaSbaaSqaaiabdEha3jabdQgaQbqabaGccqGHsislcuWGHbqygaqeamaaBaaaleaacqWG2bGDcqWGQbGAaeqaaaGccaGLhWUaayjcSdaaaa@3993@ and min(*j*) = min_*w*≠*v *_|a¯wj−a¯vj|
 MathType@MTEF@5@5@+=feaafiart1ev1aaatCvAUfKttLearuWrP9MDH5MBPbIqV92AaeXatLxBI9gBaebbnrfifHhDYfgasaacH8akY=wiFfYdH8Gipec8Eeeu0xXdbba9frFj0=OqFfea0dXdd9vqai=hGuQ8kuc9pgc9s8qqaq=dirpe0xb9q8qiLsFr0=vr0=vr0dc8meaabaqaciaacaGaaeqabaqabeGadaaakeaadaabdaqaaiqbdggaHzaaraWaaSbaaSqaaiabdEha3jabdQgaQbqabaGccqGHsislcuWGHbqygaqeamaaBaaaleaacqWG2bGDcqWGQbGAaeqaaaGccaGLhWUaayjcSdaaaa@3993@. *We have *min(*j*) ≤ *scatter *(*j*) ≤ max(*j*).

A differentially expressed gene is expected to have not only large inter-class variations, which can be represented by its *scatter*-value, but also small intra-class variations. Secondly, we define a function based on the deviation matrix *X*_*n*×*p *_and the centroid deviation matrix X¯
 MathType@MTEF@5@5@+=feaafiart1ev1aaatCvAUfKttLearuWrP9MDH5MBPbIqV92AaeXatLxBI9gBaebbnrfifHhDYfgasaacH8akY=wiFfYdH8Gipec8Eeeu0xXdbba9frFj0=OqFfea0dXdd9vqai=hGuQ8kuc9pgc9s8qqaq=dirpe0xb9q8qiLsFr0=vr0=vr0dc8meaabaqaciaacaGaaeqabaqabeGadaaakeaacuWGybawgaqeaaaa@2DFD@_*L*×*p*_:

μ(X¯L×p,j)=x^j=1L∑k=1Lx¯kj=1L∑k=1L(1nk∑i∈Ckxij),
 MathType@MTEF@5@5@+=feaafiart1ev1aaatCvAUfKttLearuWrP9MDH5MBPbIqV92AaeXatLxBI9gBaebbnrfifHhDYfgasaacH8akY=wiFfYdH8Gipec8Eeeu0xXdbba9frFj0=OqFfea0dXdd9vqai=hGuQ8kuc9pgc9s8qqaq=dirpe0xb9q8qiLsFr0=vr0=vr0dc8meaabaqaciaacaGaaeqabaqabeGadaaakeaaiiGacqWF8oqBcqGGOaakcuWGybawgaqeamaaBaaaleaacqWGmbatcqGHxdaTcqWGWbaCaeqaaOGaeiilaWIaemOAaOMaeiykaKIaeyypa0JafmiEaGNbaKaadaWgaaWcbaGaemOAaOgabeaakiabg2da9maalaaabaGaeGymaedabaGaemitaWeaamaaqahabaGafmiEaGNbaebadaWgaaWcbaGaem4AaSMaemOAaOgabeaaaeaacqWGRbWAcqGH9aqpcqaIXaqmaeaacqWGmbata0GaeyyeIuoakiabg2da9maalaaabaGaeGymaedabaGaemitaWeaamaaqahabaGaeiikaGYaaSaaaeaacqaIXaqmaeaacqWGUbGBdaWgaaWcbaGaem4AaSgabeaaaaaabaGaem4AaSMaeyypa0JaeGymaedabaGaemitaWeaniabggHiLdGcdaaeqbqaaiabdIha4naaBaaaleaacqWGPbqAcqWGQbGAaeqaaaqaaiabdMgaPjabgIGiolabdoeadnaaBaaameaacqWGRbWAaeqaaaWcbeqdcqGHris5aOGaeiykaKIaeiilaWcaaa@675D@

which is stable. Intuitively, discriminatory genes are expected to have smaller *μ*-values. In the example dataset, genes 1 and 2 have the same mean expression values across all three classes, that is, they have the equal *scatter *values. Nonetheless, *μ*(X¯
 MathType@MTEF@5@5@+=feaafiart1ev1aaatCvAUfKttLearuWrP9MDH5MBPbIqV92AaeXatLxBI9gBaebbnrfifHhDYfgasaacH8akY=wiFfYdH8Gipec8Eeeu0xXdbba9frFj0=OqFfea0dXdd9vqai=hGuQ8kuc9pgc9s8qqaq=dirpe0xb9q8qiLsFr0=vr0=vr0dc8meaabaqaciaacaGaaeqabaqabeGadaaakeaacuWGybawgaqeaaaa@2DFD@_3×4_, 1) = 0.167 and *μ*(X¯
 MathType@MTEF@5@5@+=feaafiart1ev1aaatCvAUfKttLearuWrP9MDH5MBPbIqV92AaeXatLxBI9gBaebbnrfifHhDYfgasaacH8akY=wiFfYdH8Gipec8Eeeu0xXdbba9frFj0=OqFfea0dXdd9vqai=hGuQ8kuc9pgc9s8qqaq=dirpe0xb9q8qiLsFr0=vr0=vr0dc8meaabaqaciaacaGaaeqabaqabeGadaaakeaacuWGybawgaqeaaaa@2DFD@_3×4_, 2) = 0.4, and thus gene 1 is better than gene 2 in this sense.

In the same example, we have *μ*(X¯
 MathType@MTEF@5@5@+=feaafiart1ev1aaatCvAUfKttLearuWrP9MDH5MBPbIqV92AaeXatLxBI9gBaebbnrfifHhDYfgasaacH8akY=wiFfYdH8Gipec8Eeeu0xXdbba9frFj0=OqFfea0dXdd9vqai=hGuQ8kuc9pgc9s8qqaq=dirpe0xb9q8qiLsFr0=vr0=vr0dc8meaabaqaciaacaGaaeqabaqabeGadaaakeaacuWGybawgaqeaaaa@2DFD@_3×4_, 3) = *μ*(X¯
 MathType@MTEF@5@5@+=feaafiart1ev1aaatCvAUfKttLearuWrP9MDH5MBPbIqV92AaeXatLxBI9gBaebbnrfifHhDYfgasaacH8akY=wiFfYdH8Gipec8Eeeu0xXdbba9frFj0=OqFfea0dXdd9vqai=hGuQ8kuc9pgc9s8qqaq=dirpe0xb9q8qiLsFr0=vr0=vr0dc8meaabaqaciaacaGaaeqabaqabeGadaaakeaacuWGybawgaqeaaaa@2DFD@_3×4_, 4) = 0.4. However, for gene 3, the centroids of three intra-class average deviations are the same, that is, x¯
 MathType@MTEF@5@5@+=feaafiart1ev1aaatCvAUfKttLearuWrP9MDH5MBPbIqV92AaeXatLxBI9gBaebbnrfifHhDYfgasaacH8akY=wiFfYdH8Gipec8Eeeu0xXdbba9frFj0=OqFfea0dXdd9vqai=hGuQ8kuc9pgc9s8qqaq=dirpe0xb9q8qiLsFr0=vr0=vr0dc8meaabaqaciaacaGaaeqabaqabeGadaaakeaacuWG4baEgaqeaaaa@2E3D@*k*_3 _= 0.4 for *k *= 1, 2, 3; for gene 4, the scenario is totally different, x¯
 MathType@MTEF@5@5@+=feaafiart1ev1aaatCvAUfKttLearuWrP9MDH5MBPbIqV92AaeXatLxBI9gBaebbnrfifHhDYfgasaacH8akY=wiFfYdH8Gipec8Eeeu0xXdbba9frFj0=OqFfea0dXdd9vqai=hGuQ8kuc9pgc9s8qqaq=dirpe0xb9q8qiLsFr0=vr0=vr0dc8meaabaqaciaacaGaaeqabaqabeGadaaakeaacuWG4baEgaqeaaaa@2E3D@*x*_4 _= 0.2, 0.4, 0.6 for *k *= 1, 2, 3. This raises a question of, basing on *μ*(X¯
 MathType@MTEF@5@5@+=feaafiart1ev1aaatCvAUfKttLearuWrP9MDH5MBPbIqV92AaeXatLxBI9gBaebbnrfifHhDYfgasaacH8akY=wiFfYdH8Gipec8Eeeu0xXdbba9frFj0=OqFfea0dXdd9vqai=hGuQ8kuc9pgc9s8qqaq=dirpe0xb9q8qiLsFr0=vr0=vr0dc8meaabaqaciaacaGaaeqabaqabeGadaaakeaacuWGybawgaqeaaaa@2DFD@_*L*×*p*_, *j*), what we can tell about the quality of gene *j*. The contradictory fact is that gene 3 has a smaller maximum intra-class average deviation and a larger minimum intra-class average deviation. To further differentiate the genes, thirdly, we define function *d*_1_(*j*):

d1(j)=1L∑k=1Lx¯kj2.
 MathType@MTEF@5@5@+=feaafiart1ev1aaatCvAUfKttLearuWrP9MDH5MBPbIqV92AaeXatLxBI9gBaebbnrfifHhDYfgasaacH8akY=wiFfYdH8Gipec8Eeeu0xXdbba9frFj0=OqFfea0dXdd9vqai=hGuQ8kuc9pgc9s8qqaq=dirpe0xb9q8qiLsFr0=vr0=vr0dc8meaabaqaciaacaGaaeqabaqabeGadaaakeaacqWGKbazdaWgaaWcbaGaeGymaedabeaakiabcIcaOiabdQgaQjabcMcaPiabg2da9maakaaabaWaaSaaaeaacqaIXaqmaeaacqWGmbataaWaaabCaeaacuWG4baEgaqeamaaDaaaleaacqWGRbWAcqWGQbGAaeaacqaIYaGmaaaabaGaem4AaSMaeyypa0JaeGymaedabaGaemitaWeaniabggHiLdaaleqaaOGaeiOla4caaa@427B@

From Lemma 1, *d*_1_(*j*) ≥ *μ*(X¯
 MathType@MTEF@5@5@+=feaafiart1ev1aaatCvAUfKttLearuWrP9MDH5MBPbIqV92AaeXatLxBI9gBaebbnrfifHhDYfgasaacH8akY=wiFfYdH8Gipec8Eeeu0xXdbba9frFj0=OqFfea0dXdd9vqai=hGuQ8kuc9pgc9s8qqaq=dirpe0xb9q8qiLsFr0=vr0=vr0dc8meaabaqaciaacaGaaeqabaqabeGadaaakeaacuWGybawgaqeaaaa@2DFD@_*L*×*p*_, *j*). *d*_1_(*j*) is also stable, and in the above example we have *d*_1_(3) <*d*_1_(4), which indicates that function *d*_1_(*j*) could be more sensitive and conservative than function *μ*(X¯
 MathType@MTEF@5@5@+=feaafiart1ev1aaatCvAUfKttLearuWrP9MDH5MBPbIqV92AaeXatLxBI9gBaebbnrfifHhDYfgasaacH8akY=wiFfYdH8Gipec8Eeeu0xXdbba9frFj0=OqFfea0dXdd9vqai=hGuQ8kuc9pgc9s8qqaq=dirpe0xb9q8qiLsFr0=vr0=vr0dc8meaabaqaciaacaGaaeqabaqabeGadaaakeaacuWGybawgaqeaaaa@2DFD@_*L*×*p*_, *j*) on judgment ability. Another stable function can be defined based on *μ*(X¯
 MathType@MTEF@5@5@+=feaafiart1ev1aaatCvAUfKttLearuWrP9MDH5MBPbIqV92AaeXatLxBI9gBaebbnrfifHhDYfgasaacH8akY=wiFfYdH8Gipec8Eeeu0xXdbba9frFj0=OqFfea0dXdd9vqai=hGuQ8kuc9pgc9s8qqaq=dirpe0xb9q8qiLsFr0=vr0=vr0dc8meaabaqaciaacaGaaeqabaqabeGadaaakeaacuWGybawgaqeaaaa@2DFD@_*L*×*p*_,*j*) is

d2(j)=1L∑k=1L(1nk∑i∈Ckxij2).
 MathType@MTEF@5@5@+=feaafiart1ev1aaatCvAUfKttLearuWrP9MDH5MBPbIqV92AaeXatLxBI9gBaebbnrfifHhDYfgasaacH8akY=wiFfYdH8Gipec8Eeeu0xXdbba9frFj0=OqFfea0dXdd9vqai=hGuQ8kuc9pgc9s8qqaq=dirpe0xb9q8qiLsFr0=vr0=vr0dc8meaabaqaciaacaGaaeqabaqabeGadaaakeaacqWGKbazdaWgaaWcbaGaeGOmaidabeaakiabcIcaOiabdQgaQjabcMcaPiabg2da9maakaaabaWaaSaaaeaacqaIXaqmaeaacqWGmbataaWaaabCaeaadaqadaqaamaalaaabaGaeGymaedabaGaemOBa42aaSbaaSqaaiabdUgaRbqabaaaaOWaaabuaeaacqWG4baEdaqhaaWcbaGaemyAaKMaemOAaOgabaGaeGOmaidaaaqaaiabdMgaPjabgIGiolabdoeadnaaBaaameaacqWGRbWAaeqaaaWcbeqdcqGHris5aaGccaGLOaGaayzkaaaaleaacqWGRbWAcqGH9aqpcqaIXaqmaeaacqWGmbata0GaeyyeIuoaaSqabaGccqGGUaGlaaa@4F96@

Intuitively, *d*_2_(*j*) includes more details in its calculation than *d*_1_(*j*) does. In the above example, gene 2 and gene 3 have the same mean expression values across all three classes: x¯
 MathType@MTEF@5@5@+=feaafiart1ev1aaatCvAUfKttLearuWrP9MDH5MBPbIqV92AaeXatLxBI9gBaebbnrfifHhDYfgasaacH8akY=wiFfYdH8Gipec8Eeeu0xXdbba9frFj0=OqFfea0dXdd9vqai=hGuQ8kuc9pgc9s8qqaq=dirpe0xb9q8qiLsFr0=vr0=vr0dc8meaabaqaciaacaGaaeqabaqabeGadaaakeaacuWG4baEgaqeaaaa@2E3D@_1*j *_= x¯
 MathType@MTEF@5@5@+=feaafiart1ev1aaatCvAUfKttLearuWrP9MDH5MBPbIqV92AaeXatLxBI9gBaebbnrfifHhDYfgasaacH8akY=wiFfYdH8Gipec8Eeeu0xXdbba9frFj0=OqFfea0dXdd9vqai=hGuQ8kuc9pgc9s8qqaq=dirpe0xb9q8qiLsFr0=vr0=vr0dc8meaabaqaciaacaGaaeqabaqabeGadaaakeaacuWG4baEgaqeaaaa@2E3D@_2*j *_= x¯
 MathType@MTEF@5@5@+=feaafiart1ev1aaatCvAUfKttLearuWrP9MDH5MBPbIqV92AaeXatLxBI9gBaebbnrfifHhDYfgasaacH8akY=wiFfYdH8Gipec8Eeeu0xXdbba9frFj0=OqFfea0dXdd9vqai=hGuQ8kuc9pgc9s8qqaq=dirpe0xb9q8qiLsFr0=vr0=vr0dc8meaabaqaciaacaGaaeqabaqabeGadaaakeaacuWG4baEgaqeaaaa@2E3D@_3*j*_. Therefore, we have *d*_1_(2) = *d*_1_(3) but *d*_2_(2) <*d*_2_(3). Since intuitively gene 2 has a stronger separability than gene 3, *d*_2_(*j*) could be even more sensitive than *d*_1_(*j*).

The above two functions *d*_1_(·) and *d*_2_(·) basically consider the means of intra-class variations. The following two functions *δ*_1_(*j*) and *δ*_2_(*j*) are introduced to capture the variations of intra-class deviations, corresponding to *d*_1_(·) and *d*_2_(·), respectively:

δ1(j)=1L∑k=1L(x¯kj−μ(X¯L×p,j))2,andδ2(j)=1L∑k=1L1nk∑i∈Ck(x¯ij−μ(X¯L×p,j))2.
 MathType@MTEF@5@5@+=feaafiart1ev1aaatCvAUfKttLearuWrP9MDH5MBPbIqV92AaeXatLxBI9gBaebbnrfifHhDYfgasaacH8akY=wiFfYdH8Gipec8Eeeu0xXdbba9frFj0=OqFfea0dXdd9vqai=hGuQ8kuc9pgc9s8qqaq=dirpe0xb9q8qiLsFr0=vr0=vr0dc8meaabaqaciaacaGaaeqabaqabeGadaaakeaafaqabeqadaaabaacciGae8hTdq2aaSbaaSqaaiabigdaXaqabaGccqGGOaakcqWGQbGAcqGGPaqkcqGH9aqpdaGcaaqaamaalaaabaGaeGymaedabaGaemitaWeaamaaqahabaWaaeWaaeaacuWG4baEgaqeamaaBaaaleaacqWGRbWAcqWGQbGAaeqaaOGaeyOeI0Iae8hVd0MaeiikaGIafmiwaGLbaebadaWgaaWcbaGaemitaWKaey41aqRaemiCaahabeaakiabcYcaSiabdQgaQjabcMcaPaGaayjkaiaawMcaamaaCaaaleqabaGaeGOmaidaaaqaaiabdUgaRjabg2da9iabigdaXaqaaiabdYeambqdcqGHris5aaWcbeaakiabcYcaSaqaaiabbggaHjabb6gaUjabbsgaKbqaaiab=r7aKnaaBaaaleaacqaIYaGmaeqaaOGaeiikaGIaemOAaOMaeiykaKIaeyypa0ZaaOaaaeaadaWcaaqaaiabigdaXaqaaiabdYeambaadaaeWbqaamaalaaabaGaeGymaedabaGaemOBa42aaSbaaSqaaiabdUgaRbqabaaaaOWaaabuaeaadaqadaqaaiqbdIha4zaaraWaaSbaaSqaaiabdMgaPjabdQgaQbqabaGccqGHsislcqWF8oqBcqGGOaakcuWGybawgaqeamaaBaaaleaacqWGmbatcqGHxdaTcqWGWbaCaeqaaOGaeiilaWIaemOAaOMaeiykaKcacaGLOaGaayzkaaWaaWbaaSqabeaacqaIYaGmaaaabaGaemyAaKMaeyicI4Saem4qam0aaSbaaWqaaiabdUgaRbqabaaaleqaniabggHiLdaaleaacqWGRbWAcqGH9aqpcqaIXaqmaeaacqWGmbata0GaeyyeIuoaaSqabaaaaOGaeiOla4caaa@8582@

**Theorem 4 **δ1(j)=d1(j)2−μ(X¯L×p,j)2andδ2(j)=d2(j)2−μ(X¯L×p,j)2.
 MathType@MTEF@5@5@+=feaafiart1ev1aaatCvAUfKttLearuWrP9MDH5MBPbIqV92AaeXatLxBI9gBaebbnrfifHhDYfgasaacH8akY=wiFfYdH8Gipec8Eeeu0xXdbba9frFj0=OqFfea0dXdd9vqai=hGuQ8kuc9pgc9s8qqaq=dirpe0xb9q8qiLsFr0=vr0=vr0dc8meaabaqaciaacaGaaeqabaqabeGadaaakeaafaqabeqadaaabaacciGae8hTdq2aaSbaaSqaaiabbgdaXaqabaGccqGGOaakcqWGQbGAcqGGPaqkcqGH9aqpdaGcaaqaaiabdsgaKnaaBaaaleaacqaIXaqmaeqaaOGaeiikaGIaemOAaOMaeiykaKYaaWbaaSqabeaacqaIYaGmaaGccqGHsislcqWF8oqBcqGGOaakcuWGybawgaqeamaaBaaaleaacqWGmbatcqGHxdaTcqWGWbaCaeqaaOGaeiilaWIaemOAaOMaeiykaKYaaWbaaSqabeaacqaIYaGmaaaabeaaaOqaaiabdggaHjabd6gaUjabdsgaKbqaaiab=r7aKnaaBaaaleaacqqGYaGmaeqaaOGaeiikaGIaemOAaOMaeiykaKIaeyypa0ZaaOaaaeaacqWGKbazdaWgaaWcbaGaeGOmaidabeaakiabcIcaOiabdQgaQjabcMcaPmaaCaaaleqabaGaeGOmaidaaOGaeyOeI0Iae8hVd0MaeiikaGIafmiwaGLbaebadaWgaaWcbaGaemitaWKaey41aqRaemiCaahabeaakiabcYcaSiabdQgaQjabcMcaPmaaCaaaleqabaGaeGOmaidaaaqabaaaaOGaeiOla4caaa@6892@

PROOF. The proof is easily done by simplifying the definition formulae for *δ*_1_(*j*) and *δ*_2_(*j*).     □

Similar to functions *d*_1_(*j*) and *d*_2_(*j*), for an ideal differentially expressed gene *j*, both *δ*_1_(*j*) and *δ*_2_(*j*) are expected to have small values. Moreover, similar to the relation between *d*_1_(*j*) and *d*_2_(*j*), *δ*_2_(*j*) is considered more sensitive than *δ*_1_(*j*). We define function *compact*_*k*_(*j*) = *d*_*k*_(*j*) + *δ*_*k*_(*j*), for *k *= 1, 2, to evaluate the intra-class variations for gene *j*. And we define the gene scoring function *s*_*k*_(*j*) = *compact*_*k*_(*j*)/*scatter*(*j*) to rank the genes according to their differentiability. Note that a smaller value of *s*_*k*_(*j*) indicates a higher differentiability.

We denote the gene selection method using *compact*_1_(*j*) = *d*_1_(*j*) + *δ*_1_(*j*) as GS1, and the other using *compact*_2_(*j*) = *d*_2_(*j*) + *δ*_2_(*j*) as GS2. Both GS1 and GS2 are model-free and stable. In each of them, the scores for all genes are calculated and genes are sorted in non-decreasing order of their scores. Since the number of genes, *p*, is typically much larger than the number of samples, *n*, the overall running time to compute this order is *O*(*p *log *p*). In practice, there are several ways to select the informative genes using this order. For example, one may select the top ranked *x *genes for further analysis, or the top ranked *x% *genes, or all the genes with score no larger than some constant, among others.

### F-test method

F-test method [1,19] is also a single gene scoring approach. Besides the notations used in our methods, it uses σk2
 MathType@MTEF@5@5@+=feaafiart1ev1aaatCvAUfKttLearuWrP9MDH5MBPbIqV92AaeXatLxBI9gBaebbnrfifHhDYfgasaacH8akY=wiFfYdH8Gipec8Eeeu0xXdbba9frFj0=OqFfea0dXdd9vqai=hGuQ8kuc9pgc9s8qqaq=dirpe0xb9q8qiLsFr0=vr0=vr0dc8meaabaqaciaacaGaaeqabaqabeGadaaakeaaiiGacqWFdpWCdaqhaaWcbaGaem4AaSgabaGaeGOmaidaaaaa@30F4@ to denote the variance of expression value of gene *j *in the *k*-th class:

σk2=∑i∈Ck(aij−a¯kj)2nk−1,
 MathType@MTEF@5@5@+=feaafiart1ev1aaatCvAUfKttLearuWrP9MDH5MBPbIqV92AaeXatLxBI9gBaebbnrfifHhDYfgasaacH8akY=wiFfYdH8Gipec8Eeeu0xXdbba9frFj0=OqFfea0dXdd9vqai=hGuQ8kuc9pgc9s8qqaq=dirpe0xb9q8qiLsFr0=vr0=vr0dc8meaabaqaciaacaGaaeqabaqabeGadaaakeaaiiGacqWFdpWCdaqhaaWcbaGaem4AaSgabaGaeGOmaidaaOGaeyypa0ZaaSaaaeaadaaeqaqaaiabcIcaOiabdggaHnaaBaaaleaacqWGPbqAcqWGQbGAaeqaaOGaeyOeI0IafmyyaeMbaebadaWgaaWcbaGaem4AaSMaemOAaOgabeaakiabcMcaPmaaCaaaleqabaGaeGOmaidaaaqaaiabdMgaPjabgIGiolabdoeadnaaBaaameaacqWGRbWAaeqaaaWcbeqdcqGHris5aaGcbaGaemOBa42aaSbaaSqaaiabdUgaRbqabaGccqGHsislcqaIXaqmaaGaeiilaWcaaa@4B7E@

and σ2=∑k=1Lnk(nk−1)σk2n−L
 MathType@MTEF@5@5@+=feaafiart1ev1aaatCvAUfKttLearuWrP9MDH5MBPbIqV92AaeXatLxBI9gBaebbnrfifHhDYfgasaacH8akY=wiFfYdH8Gipec8Eeeu0xXdbba9frFj0=OqFfea0dXdd9vqai=hGuQ8kuc9pgc9s8qqaq=dirpe0xb9q8qiLsFr0=vr0=vr0dc8meaabaqaciaacaGaaeqabaqabeGadaaakeaaiiGacqWFdpWCdaahaaWcbeqaaiabikdaYaaakiabg2da9maalaaabaWaaabmaeaacqWGUbGBdaWgaaWcbaGaem4AaSgabeaakiabcIcaOiabd6gaUnaaBaaaleaacqWGRbWAaeqaaOGaeyOeI0IaeGymaeJaeiykaKIae83Wdm3aa0baaSqaaiabdUgaRbqaaiabikdaYaaaaeaacqWGRbWAcqGH9aqpcqaIXaqmaeaacqWGmbata0GaeyyeIuoaaOqaaiabd6gaUjabgkHiTiabdYeambaaaaa@485E@ to denote the variance in the whole dataset. Gene *j *has a score defined to be:

F(j)=∑k=1Lnk(a¯kj−a^j)/(L−1)σ2
 MathType@MTEF@5@5@+=feaafiart1ev1aaatCvAUfKttLearuWrP9MDH5MBPbIqV92AaeXatLxBI9gBaebbnrfifHhDYfgasaacH8akY=wiFfYdH8Gipec8Eeeu0xXdbba9frFj0=OqFfea0dXdd9vqai=hGuQ8kuc9pgc9s8qqaq=dirpe0xb9q8qiLsFr0=vr0=vr0dc8meaabaqaciaacaGaaeqabaqabeGadaaakeaacqWGgbGrcqGGOaakcqWGQbGAcqGGPaqkcqGH9aqpdaWcaaqaamaaqadabaGaemOBa42aaSbaaSqaaiabdUgaRbqabaGccqGGOaakcuWGHbqygaqeamaaBaaaleaacqWGRbWAcqWGQbGAaeqaaOGaeyOeI0IafmyyaeMbaKaadaWgaaWcbaGaemOAaOgabeaakiabcMcaPiabc+caViabcIcaOiabdYeamjabgkHiTiabigdaXiabcMcaPaWcbaGaem4AaSMaeyypa0JaeGymaedabaGaemitaWeaniabggHiLdaakeaaiiGacqWFdpWCdaahaaWcbeqaaiabikdaYaaaaaaaaa@4DC3@

### Cho's method

Using the same notations used as in the above, Cho's method [17] defines a weight factor *w*_*i *_for sample *i*, which is 1nk
 MathType@MTEF@5@5@+=feaafiart1ev1aaatCvAUfKttLearuWrP9MDH5MBPbIqV92AaeXatLxBI9gBaebbnrfifHhDYfgasaacH8akY=wiFfYdH8Gipec8Eeeu0xXdbba9frFj0=OqFfea0dXdd9vqai=hGuQ8kuc9pgc9s8qqaq=dirpe0xb9q8qiLsFr0=vr0=vr0dc8meaabaqaciaacaGaaeqabaqabeGadaaakeaadaWcaaqaaiabigdaXaqaaiabd6gaUnaaBaaaleaacqWGRbWAaeqaaaaaaaa@309C@ if sample *i *belongs to class *k*. Let W=∑i=1nwi
 MathType@MTEF@5@5@+=feaafiart1ev1aaatCvAUfKttLearuWrP9MDH5MBPbIqV92AaeXatLxBI9gBaebbnrfifHhDYfgasaacH8akY=wiFfYdH8Gipec8Eeeu0xXdbba9frFj0=OqFfea0dXdd9vqai=hGuQ8kuc9pgc9s8qqaq=dirpe0xb9q8qiLsFr0=vr0=vr0dc8meaabaqaciaacaGaaeqabaqabeGadaaakeaacqWGxbWvcqGH9aqpdaaeWaqaaiabdEha3naaBaaaleaacqWGPbqAaeqaaaqaaiabdMgaPjabg2da9iabigdaXaqaaiabd6gaUbqdcqGHris5aaaa@3894@. The weighted *mean*(*j*) for gene *j *is defined as

mean(j)=∑i=1nwiWxij.
 MathType@MTEF@5@5@+=feaafiart1ev1aaatCvAUfKttLearuWrP9MDH5MBPbIqV92AaeXatLxBI9gBaebbnrfifHhDYfgasaacH8akY=wiFfYdH8Gipec8Eeeu0xXdbba9frFj0=OqFfea0dXdd9vqai=hGuQ8kuc9pgc9s8qqaq=dirpe0xb9q8qiLsFr0=vr0=vr0dc8meaabaqaciaacaGaaeqabaqabeGadaaakeaacqWGTbqBcqWGLbqzcqWGHbqycqWGUbGBcqGGOaakcqWGQbGAcqGGPaqkcqGH9aqpdaaeWbqaamaalaaabaGaem4DaC3aaSbaaSqaaiabdMgaPbqabaaakeaacqWGxbWvaaGaemiEaG3aaSbaaSqaaiabdMgaPjabdQgaQbqabaaabaGaemyAaKMaeyypa0JaeGymaedabaGaemOBa4ganiabggHiLdGccqGGUaGlaaa@46AE@

The weighted standard deviation is defined as

std(j)=∑i=1n(xij−mean(j))2(n−1/n)∑i=1nwi.
 MathType@MTEF@5@5@+=feaafiart1ev1aaatCvAUfKttLearuWrP9MDH5MBPbIqV92AaeXatLxBI9gBaebbnrfifHhDYfgasaacH8akY=wiFfYdH8Gipec8Eeeu0xXdbba9frFj0=OqFfea0dXdd9vqai=hGuQ8kuc9pgc9s8qqaq=dirpe0xb9q8qiLsFr0=vr0=vr0dc8meaabaqaciaacaGaaeqabaqabeGadaaakeaacqWGZbWCcqWG0baDcqWGKbazcqGGOaakcqWGQbGAcqGGPaqkcqGH9aqpdaGcaaqaamaalaaabaWaaabmaeaacqGGOaakcqWG4baEdaWgaaWcbaGaemyAaKMaemOAaOgabeaakiabgkHiTiabd2gaTjabdwgaLjabdggaHjabd6gaUjabcIcaOiabdQgaQjabcMcaPiabcMcaPmaaCaaaleqabaGaeGOmaidaaaqaaiabdMgaPjabg2da9iabigdaXaqaaiabd6gaUbqdcqGHris5aaGcbaGaeiikaGIaemOBa4MaeyOeI0IaeGymaeJaei4la8IaemOBa4MaeiykaKYaaabmaeaacqWG3bWDdaWgaaWcbaGaemyAaKgabeaaaeaacqWGPbqAcqGH9aqpcqaIXaqmaeaacqWGUbGBa0GaeyyeIuoaaaaaleqaaOGaeiOla4caaa@5E46@

Then the score of gene *j *is calculated as

C(j)=mean(j)×std(j)std(a¯j),
 MathType@MTEF@5@5@+=feaafiart1ev1aaatCvAUfKttLearuWrP9MDH5MBPbIqV92AaeXatLxBI9gBaebbnrfifHhDYfgasaacH8akY=wiFfYdH8Gipec8Eeeu0xXdbba9frFj0=OqFfea0dXdd9vqai=hGuQ8kuc9pgc9s8qqaq=dirpe0xb9q8qiLsFr0=vr0=vr0dc8meaabaqaciaacaGaaeqabaqabeGadaaakeaacqWGdbWqcqGGOaakcqWGQbGAcqGGPaqkcqGH9aqpdaWcaaqaaiabd2gaTjabdwgaLjabdggaHjabd6gaUjabcIcaOiabdQgaQjabcMcaPiabgEna0kabdohaZjabdsha0jabdsgaKjabcIcaOiabdQgaQjabcMcaPaqaaiabdohaZjabdsha0jabdsgaKjabcIcaOiqbdggaHzaaraWaaSbaaSqaaiabdQgaQbqabaGccqGGPaqkaaGaeiilaWcaaa@4D65@

where *std*(a¯j
 MathType@MTEF@5@5@+=feaafiart1ev1aaatCvAUfKttLearuWrP9MDH5MBPbIqV92AaeXatLxBI9gBaebbnrfifHhDYfgasaacH8akY=wiFfYdH8Gipec8Eeeu0xXdbba9frFj0=OqFfea0dXdd9vqai=hGuQ8kuc9pgc9s8qqaq=dirpe0xb9q8qiLsFr0=vr0=vr0dc8meaabaqaciaacaGaaeqabaqabeGadaaakeaacuWGHbqygaqeamaaBaaaleaacqWGQbGAaeqaaaaa@2F98@) is the standard deviation of centroid expression values (a¯ij,a¯2j,…,a¯Lj
 MathType@MTEF@5@5@+=feaafiart1ev1aaatCvAUfKttLearuWrP9MDH5MBPbIqV92AaeXatLxBI9gBaebbnrfifHhDYfgasaacH8akY=wiFfYdH8Gipec8Eeeu0xXdbba9frFj0=OqFfea0dXdd9vqai=hGuQ8kuc9pgc9s8qqaq=dirpe0xb9q8qiLsFr0=vr0=vr0dc8meaabaqaciaacaGaaeqabaqabeGadaaakeaacuWGHbqygaqeamaaBaaaleaacqWGPbqAcqWGQbGAaeqaaOGaeiilaWIafmyyaeMbaebadaWgaaWcbaGaeGOmaiJaemOAaOgabeaakiabcYcaSiablAciljabcYcaSiqbdggaHzaaraWaaSbaaSqaaiabdYeamjabdQgaQbqabaaaaa@3CB4@).

## Authors' contributions

KY and ZC contributed equally to this work. They both participated in the design of the scoring functions and their implementations. ZC, JL, and GL drafted the manuscript. GL designed the framework, supervised the whole work, and finalized the manuscript. All authors read and approved the final manuscript.

## Supplementary Material

Additional File 1 Plots of the LOO and 5-fold cross validation classification accuracies of the SVM-classifier and the KNN-classifier built on the top ranked *x *genes, for *x *≤ 100, on all eight datasets LEU, SRBCT, GLIOMA, CAR, LUNG, MLL, PROSTATE, and DLBCL. One plot corresponds to a type of cross validation classification accuracies of one classifier combined with each of the four gene selection methods on one dataset. There are in total 32 plots. Click here for file

Additional File 2 Plots of standard deviations for the 5-fold cross validation classification accuracies. One plot corresponds to the two classifiers combined with a gene selection method. There are in total 4 plots. Click here for file

Additional File 3 Four microarray datasets (CAR, DLBCL, GLIOMA, and LEU) in MATLAB format, which are used in this work. Click here for file

Additional File 4 Four microarray datasets (LUNG, MLL, PROSTATE, and SRBCT) in MATLAB format, which are used in this work. Click here for file

Additional File 5 MATLAB codes for gene selection methods GS1 and GS2. Click here for file

## Figures and Tables

**Table 1:** The leave-one-out and 5-fold cross validation classification accuracies of the SVM-classifier and the KNN-classifier based on four gene selection methods, GS1, GS2, Cho's, and F-test, on the SRBCT dataset. Listed are the accuracies when the numbers of selected genes are 30, 60, and 100, respectively, together with their standard deviations for 5-fold cross validation, and the best accuracy together with the number of selected genes.

SRBCT 5-Fold	KNN				SVMs			
	
	30	60	100	Best Accuracy/# Genes	30	60	100	Best Accuracy/# Genes
GS2	0.953 ± 0.048	0.971 ± 0.041	0.980 ± 0.038	0.981/90	0.949 ± 0.047	0.976 ± 0.040	0.990 ± 0.026	0.990/99
GS1	0.941 ± 0.047	0.961 ± 0.045	0.977 ± 0.041	0.980/88	0.959 ± 0.054	0.978 ± 0.040	0.988 ± 0.030	0.979/93
Cho's	0.820 ± 0.096	0.864 ± 0.093	0.896 ± 0.087	0.902/98	0.835 ± 0.088	0.918 ± 0.069	0.943 ± 0.062	0.943/98
F-test	0.963 ± 0.050	0.973 ± 0.046	0.978 ± 0.040	0.980/90	0.970 ± 0.042	0.980 ± 0.039	0.992 ± 0.021	0.992/95

SRBCT LOO	KNN				SVMs			
	
	30	60	100	Best Accuracy/#Genes	30	60	100	Best Accuracy/# Genes

GS2	0.964	0.976	0.964	0.988/77	0.952	0.976	1.000	1.000/96
GS1	0.964	0.988	0.988	0.988/57	0.976	0.988	0.988	0.988/34
Cho's	0.831	0.880	0.892	0.928/82	0.819	0.928	0.964	0.988/80
F-test	0.976	0.976	0.988	0.988/89	0.976	0.980	0.988	1.000/78

**Table 2:** The leave-one-out and 5-fold cross validation classification accuracies of the SVM-classifier and the KNN-classifier based on four gene selection methods, GS1, GS2, Cho's, and F-test, on the CAR dataset.

CAR 5-Fold	KNN				SVMs			
	
	30	60	100	Best Accuracy/# Genes	30	60	100	Best Accuracy/# Genes
GS2	0.578 ± 0.118	0.810 ± 0.084	0.865 ± 0.059	0.865/100	0.528 ± 0.116	0.812 ± 0.080	0.870 ± 0.053	0.870/100
GS1	0.634 ± 0.136	0.831 ± 0.079	0.874 ± 0.058	0.874/100	0.600 ± 0.140	0.824 ± 0.076	0.885 ± 0.050	0.885/100
Cho's	0.471 ± 0.091	0.676 ± 0.083	0.797 ± 0.070	0.797/100	0.437 ± 0.089	0.651 ± 0.085	0.821 ± 0.066	0.821/100
F-test	0.681 ± 0.091	0.788 ± 0.071	0.851 ± 0.065	0.851/100	0.649 ± 0.093	0.802 ± 0.071	0.868 ± 0.056	0.868/100

CAR LOO	KNN				SVMs			
	
	30	60	100	Best Accuracy/#Genes	30	60	100	Best Accuracy/# Genes

GS2	0.621	0.828	0.885	0.885/99	0.557	0.822	0.868	0.874/71
GS1	0.718	0.822	0.868	0.879/97	0.695	0.828	0.902	0.902/100
Cho's	0.448	0.661	0.787	0.805/88	0.466	0.661	0.851	0.879/97
F-test	0.707	0.776	0.856	0.862/85	0.626	0.793	0.874	0.885/97

**Table 3:** The leave-one-out and 5-fold cross validation classification accuracies of the SVM-classifier and the KNN-classifier based on four gene selection methods, GS1, GS2, Cho's, and F-test, on the LEU dataset.

LEU 5-Fold	KNN				SVMs			
	
	30	60	100	Best Accuracy/#Genes	30	60	100	Best Accuracy/#Genes
GS2	0.961 ± 0.048	0.968 ± 0.044	0.971 ± 0.040	0.971/85	0.958 ± 0.052	0.967 ± 0.047	0.974 ± 0.039	0.974/98
GS1	0.965 ± 0.048	0.973 ± 0.040	0.979 ± 0.034	0.979/100	0.965 ± 0.050	0.970 ± 0.043	0.979 ± 0.037	0.979/93
Cho's	0.958 ± 0.049	0.963 ± 0.046	0.968 ± 0.043	0.968/100	0.953 ± 0.054	0.962 ± 0.053	0.970 ± 0.043	0.970/98
F-test	0.960 ± 0.049	0.966 ± 0.045	0.974 ± 0.038	0.974/96	0.957 ± 0.055	0.968 ± 0.049	0.975 ± 0.039	0.975/99

LEU LOO	KNN				SVMs			
	
	30	60	100	Best Accuracy/#Genes	30	60	100	Best Accuracy/# Genes

GS2	0.944	0.972	0.958	0.986/10	0.958	0.958	0.972	0.986/25
GS1	0.958	0.986	0.972	0.986/60	0.972	0.986	0.986	0.986/4
Cho's	0.944	0.944	0.958	0.972/9	0.958	0.958	0.986	0.986/80
F-test	0.944	0.944	0.972	0.986/25	0.958	0.958	0.972	0.986/33

**Table 4:** The leave-one-out and 5-fold cross validation classification accuracies of the SVM-classifier and the KNN-classifier based on four gene selection methods, GS1, GS2, Cho's, and F-test, on the GLIOMA dataset.

GLIOMA 5-Fold	KNN				SVMs			
	
	30	60	100	Best Accuracy/# Genes	30	60	100	Best Accuracy/# Genes
GS2	0.660 ± 0.141	0.670 ± 0.140	0.671 ± 0.140	0.676/90	0.651 ± 0.134	0.679 ± 0.133	0.699 ± 0.131	0.701/97
GS1	0.674 ± 0.143	0.677 ± 0.148	0.679 ± 0.141	0.684/97	0.659 ± 0.145	0.698 ± 0.137	0.720 ± 0.138	0.722/99
Cho's	0.659 ± 0.145	0.660 ± 0.141	0.652 ± 0.131	0.664/31	0.618 ± 0.141	0.662 ± 0.131	0.668 ± 0.132	0.670/96
F-test	0.647 ± 0.140	0.663 ± 0.142	0.667 ± 0.133	0.674/91	0.639 ± 0.138	0.672 ± 0.131	0.684 ± 0.130	0.685/84

GLIOMA LOO	KNN				SVMs			
	
	30	60	100	Best Accuracy/#Genes	30	60	100	Best Accuracy/# Genes

GS2	0.760	0.700	0.660	0.780/28	0.700	0.660	0.760	0.760/96
GS1	0.700	0.760	0.740	0.780/35	0.680	0.700	0.760	0.760/45
Cho's	0.720	0.640	0.640	0.820/20	0.640	0.680	0.620	0.720/2
F-test	0.700	0.660	0.700	0.780/70	0.640	0.620	0.740	0.740/100

**Table 5:** The leave-one-out and 5-fold cross validation classification accuracies of the SVM-classifier and the KNN-classifier based on four gene selection methods, GS1, GS2, Cho's, and F-test, on the LUNG dataset.

LUNG 5-Fold	KNN				SVMs			
	
	30	60	100	Best Accuracy/#Genes	30	60	100	Best Accuracy/#Genes
GS2	0.884 ± 0.053	0.916 ± 0.041	0.928 ± 0.037	0.928/100	0.858 ± 0.061	0.913 ± 0.035	0.931 ± 0.033	0.931/99
GS1	0.890 ± 0.046	0.919 ± 0.041	0.937 ± 0.034	0.937/99	0.871 ± 0.051	0.922 ± 0.038	0.938 ± 0.031	0.938/98
Cho's	0.843 ± 0.053	0.897 ± 0.044	0.924 ± 0.038	0.924/100	0.803 ± 0.065	0.894 ± 0.044	0.924 ± 0.035	0.924/100
F-test	0.873 ± 0.049	0.882 ± 0.044	0.918 ± 0.044	0.918/100	0.852 ± 0.055	0.901 ± 0.042	0.930 ± 0.036	0.930/100

LUNG LOO	KNN				SVMs			
	
	30	60	100	Best Accuracy/# Genes	30	60	100	Best Accuracy/# Genes

GS2	0.892	0.906	0.921	0.931/44	0.867	0.892	0.931	0.931/73
GS1	0.887	0.941	0.941	0.951/49	0.862	0.941	0.941	0.951/51
Cho's	0.837	0.897	0.921	0.926/86	0.773	0.892	0.931	0.941/88
F-test	0.872	0.877	0.901	0.921/89	0.857	0.901	0.926	0.936/94

**Table 6:** The leave-one-out and 5-fold cross validation classification accuracies of the SVM-classifier and the KNN-classifier based on four gene selection methods, GS1, GS2, Cho's, and F-test, on the DLBCL dataset.

DLBCL 5-Fold	KNN				SVMs			
	
	30	60	100	Best Accuracy/# Genes	30	60	100	Best Accuracy/# Genes
GS2	0.881 ± 0.081	0.906 ± 0.078	0.914 ± 0.074	0.916/98	0.872 ± 0.081	0.918 ± 0.068	0.933 ± 0.054	0.933/98
GS1	0.878 ± 0.078	0.895 ± 0.075	0.903 ± 0.076	0.903/100	0.861 ± 0.079	0.895 ± 0.075	0.917 ± 0.066	0.918/98
Cho's	0.874 ± 0.085	0.909 ± 0.075	0.920 ± 0.072	0.920/99	0.869 ± 0.085	0.915 ± 0.068	0.930 ± 0.061	0.930/99
F-test	0.877 ± 0.079	0.893 ± 0.078	0.902 ± 0.080	0.902/100	0.869 ± 0.079	0.910 ± 0.074	0.925 ± 0.063	0.926/95

DLBCL LOO	KNN				SVMs			
	
	30	60	100	Best Accuracy/# Genes	30	60	100	Best Accuracy/# Genes

GS2	0.883	0.922	0.922	0.935/70	0.896	0.961	0.948	0.961/55
GS1	0.896	0.896	0.922	0.922/85	0.870	0.883	0.961	0.961/81
Cho's	0.883	0.922	0.922	0.935/69	0.909	0.896	0.948	0.961/74
F-test	0.896	0.896	0.883	0.922/61	0.857	0.935	0.948	0.961/92

**Table 7:** The leave-one-out and 5-fold cross validation classification accuracies of the SVM-classifier and the KNN-classifier based on four gene selection methods, GS1, GS2, Cho's, and F-test, on the MLL dataset.

MLL 5-Fold	KNN				SVMs			
	
	30	60	100	Best Accuracy/#Genes	30	60	100	Best Accuracy/#Genes
GS2	0.937 ± 0.056	0.947 ± 0.055	0.948 ± 0.053	0.949/91	0.926 ± 0.058	0.941 ± 0.052	0.947 ± 0.051	0.947/87
GS1	0.946 ± 0.054	0.940 ± 0.057	0.942 ± 0.058	0.948/29	0.932 ± 0.059	0.947 ± 0.053	0.952 ± 0.050	0.952/99
Cho's	0.950 ± 0.048	0.954 ± 0.048	0.960 ± 0.045	0.960/93	0.942 ± 0.051	0.946 ± 0.050	0.955 ± 0.048	0.955/89
F-test	0.949 ± 0.050	0.950 ± 0.050	0.953 ± 0.051	0.954/99	0.943 ± 0.051	0.945 ± 0.053	0.948 ± 0.051	0.948/100

MLL LOO	KNN				SVMs			
	
	30	60	100	Best Accuracy/# Genes	30	60	100	Best Accuracy/# Genes

GS2	0.944	0.958	0.972	0.972/90	0.917	0.958	0.944	0.972/91
GS1	0.958	0.944	0.958	0.972/97	0.958	0.958	0.958	0.972/56
Cho's	0.944	0.944	0.958	0.972/23	0.944	0.931	0.944	0.958/44
F-test	0.944	0.944	0.958	0.958/65	0.944	0.931	0.944	0.958/31

**Table 8:** The leave-one-out and 5-fold cross validation classification accuracies of the SVM-classifier and the KNN-classifier based on four gene selection methods, GS1, GS2, Cho's, and F-test, on the PROSTATE dataset.

PROSTATE 5-Fold	KNN				SVMs			
	
	30	60	100	Best Accuracy/# Genes	30	60	100	Best Accuracy/#Genes
GS2	0.917 ± 0.073	0.916 ± 0.056	0.913 ± 0.057	0.921/39	0.884 ± 0.080	0.908 ± 0.057	0.909 ± 0.060	0.911/91
GS1	0.918 ± 0.073	0.917 ± 0.056	0.907 ± 0.062	0.922/35	0.887 ± 0.082	0.901 ± 0.060	0.914 ± 0.058	0.914/99
Cho's	0.870 ± 0.144	0.918 ± 0.055	0.914 ± 0.058	0.918/10	0.841 ± 0.149	0.890 ± 0.069	0.904 ± 0.061	0.904/4
F-test	0.921 ± 0.053	0.915 ± 0.056	0.913 ± 0.057	0.935/61	0.893 ± 0.060	0.907 ± 0.062	0.914 ± 0.058	0.918/92

PROSTATE LOO	KNN				SVMs			
	
	30	60	100	Best Accuracy/# Genes	30	60	100	Best Accuracy/#Genes

GS2	0.931	0.922	0.922	0.941/8	0.902	0.902	0.941	0.951/47
GS1	0.931	0.922	0.902	0.951/8	0.931	0.912	0.922	0.951/49
Cho's	0.931	0.912	0.912	0.941/8	0.941	0.912	0.912	0.941/20
F-test	0.931	0.922	0.922	0.941/10	0.892	0.931	0.931	0.941/4

**Table 9:** The percentages of genes that were re-selected by Cho's and F-test on duplicated datasets, of the whole LEU and the SRBCT datasets, respectively.

Method	*x*	Whole Dataset					
		
		SRBCT				LEU	
		
		EWS	BL	NB	RMS	ALL	AML
Cho's	30	90.0%	93.3%	90.0%	86.7%	96.7%	83.0%
	74	90.5%	89.2%	91.9%	91.9%	93.2%	86.5%
	100	90.0%	94.0%	92.0%	91.0%	92.0%	90.0%
F-test	30	90.7%	85.3%	86.7%	89.3%	83.3%	83.3%
	74	90.7%	85.3%	86.7%	83.3%	86.7%	89.3%
	100	89.0%	87.0%	88.0%	86.0%	92.0%	87.0%

**Table 10:** The percentages of genes that were re-selected by GS1, GS2, Cho's, and F-test on reduced datasets, of the whole LEU and the SRBCT datasets, respectively.

*x*	Method	Whole Dataset					
		
		SRBCT				LEU	
		
		EWS	BL	NB	RMS	ALL	AML
30	GS2	87.5%	81.3%	85.0%	83.8%	87.4%	84.9%
	GS1	82.9%	73.9%	80.4%	81.8%	85.5%	80.4%
	Cho's	83.2%	79.9%	85.5%	83.4%	75.0%	79.0%
	F-test	92.2%	92.1%	90.8%	93.3%	84.4%	80.1%
74	GS2	85.5%	82.6%	86.4%	83.9%	84.5%	80.8%
	GS1	84.6%	80.2%	84.3%	85.2%	83.0%	80.9%
	Cho's	87.5%	86.9%	88.3%	85.0%	75.8%	77.0%
	F-test	87.6%	92.2%	89.3%	88.0%	83.6%	80.7%
100	GS2	86.6%	83.9%	87.6%	86.3%	83.7%	80.8%
	GS1	84.9%	79.2%	84.1%	82.9%	83.6%	80.9%
	Cho's	88.7%	84.5%	89.5%	86.0%	77.4%	75.8%
	F-test	89.3%	92.0%	89.5%	89.2%	83.0%	83.9%
